# Functional divergence of the NIP III subgroup proteins involved altered selective constraints and positive selection

**DOI:** 10.1186/1471-2229-10-256

**Published:** 2010-11-20

**Authors:** Qingpo Liu, Zhujun Zhu

**Affiliations:** 1 College of Agriculture and Food Science, Zhejiang A & F University, Lin'an, Hangzhou 311300, China

## Abstract

**Background:**

Nod26-like intrinsic proteins (NIPs) that belong to the aquaporin superfamily are unique to plants. According to homology modeling and phylogenetic analysis, the NIP subfamily can be further divided into three subgroups with distinct biological functions (NIP I, NIP II, and NIP III). In some grasses, the NIP III subgroup proteins (NIP2s) were demonstrated to be permeable to solutes with larger diameter, such as silicic acid and arsenous acids. However, to date there is no data-mining or direct experimental evidences for the permeability of such larger solutes for dicot NIP2s, although they exhibit similar three-dimensional structures as those in grasses. It is therefore intriguing to investigate the molecular mechanisms that drive the evolution of plant NIP2s.

**Results:**

The NIP III subgroup is more ancient with a divergence time that predates the monocot-dicot split. The proliferation of *NIP2 *genes in modern grass species is primarily attributed to whole genome and segmental chromosomal duplication events. The structure of *NIP2 *genes is relatively conserved, possessing five exons and four introns. All NIP2s possess an ar/R filter consisting of G, S, G, and R, except for the cucumber CsNIP2;2, where a small G in the H2 is substituted with the bulkier C residue. Our maximum likelihood analysis revealed that NIP2s, especially the loop A (LA) region, have undergone strong selective pressure for adaptive evolution. The analysis at the amino acid level provided strong statistical evidences for the functional divergence between monocot and dicot NIP III subgroup proteins. In addition, several SDPs (Specificity Determining Positions) responsible for functional specificity were predicted.

**Conclusions:**

The present study provides the first evidences of functional divergence between dicot and monocot *NIP2s*, and suggests that positive selection, as well as a radical shift of evolutionary rate at some critical amino acid sites is the primary driver. These findings will expand our understanding to evolutionary mechanisms driving the functional diversification of monocot and dicot NIP III subgroup proteins.

## Background

NOD26-like intrinsic proteins (NIPs) are plant specific integral membrane proteins, belonging to the aquaporin water channel superfamily. NIPs can be traced back to the early developmental stage of primitive land plants [1], indicating an important role during their evolution. Amongst plant aquaporins, only NIPs have the glycerol transport activity [2]. It was reported that there are 9, 13, 9 and 6 *NIP *genes encoded in the *Arabidopsis*, rice, sorghum, and *Cucumis sativus *genomes, respectively [1]. The divergence and proliferation of *NIPs *may be an adaptive response to an ever-changing environment [3].

Recent experimental evidences suggest that NIPs could perform a diverse range of functions, including a wider range for selectivity [4,5]. It was demonstrated that two constriction points within the pore, referred to as the conserved dual NPA motifs and the aromatic/arginine (ar/R) selectivity filter, primarily determine the substrate selectivity of plant aquaporins [4]. However, it appears that the ar/R filter, which consists of four amino acid residues from helix H2, helix H5, and loop LE1 + LE2, plays a crucial role in determining the substrate selectivity of NIPs [6]. Based on difference in the ar/R filter motif and on our phylogenetic tree reconstruction, it is evident that NIPs should be divided into three distinct subgroups [1,7]. The properties of the four residues making up the ar/R filter are remarkably different, leading to the significant variation of permeation ability for the proteins within each subgroup. In comparison to the NIP I and NIP II subgroups, NIP III proteins possess the largest constriction size of the pore (≥6Å), which allows larger solutes such as silicic acid (diameter 4.38Å) to permeate [6,8].

With a few exceptions, the function of most of the NIP III subgroup proteins remains unknown. Of plant aquaporins, rice genes OsNIP2;1 (Lsi1) and OsNIP2;2 (Lsi6), and barley HvNIP2;1 (HvLsi1), which belong to the NIP III subgroup and localize in the plasma membrane, were demonstrated to function as a transporter of silicon across the biomembrane [9-11], a compound that can enhance the resistance of plants to biotic and abiotic stress. Moreover, it was found that rice OsNIP2;1 could be permeable to water, urea, boric acid, and silicic acid, but not glycerol. However, competition experiments indicated that this gene is a highly specific transporter of silicic acid [6]. When expressed in oocytes, both OsNIP2;1 and OsNIP2;2 were shown to have transport activity for arsenite but not arsenate [12,13], with the former gene having a greater role in the major pathway for arsenite uptake [13]. In addition, the rice OsNIP2;1 was found to be responsible, at least partly, for the permeability to methylated arsenic species MMA and DMA [14]. All three arsenous acids are slightly smaller than silicic acid [13,14], which may explain the permeability of OsNIP2;1. Given that arsenite and glycerol are structurally similar [12], it is surprising that rice OsNIP2;1 does not transport glycerol [6,13], and suggests that other structural features may be involved in the process of efficient substrate discrimination [6,12].

*NIP *genes generally exhibit a low expression level, and often show a tissue- and/or cell type specific expression pattern [1,15,16]. The rice gene *OsNIP2;1 *is specifically expressed in roots, and the expression level can be transiently enhanced during the heading stage [10,17]; whereas *OsNIP2;2 *is expressed in both the shoots and roots, and its expression level in roots is much weaker than that of *OsNIP2;1 *[11]. The difference in expression pattern is consistent with their distinct silicon transport functions [10,11]. Moreover, it was observed that the accumulation of silicon in different rice cultivars varies extensively, which might result from the differences in the expression of *OsNIP2;1 *and other silicon transporter genes in rice roots [17]. The barley *HvLsi1*, like rice *OsNIP2;1*, is specifically expressed in the basal root, and a weak correlation between silicon uptake and the expression level of *HvLsi1 *in eight tested cultivars was observed [9]. Moshelion et al. [18] examined the expression pattern of 33 aquaporin genes using macro-array hybridization, and found that *ZmNIP2;1 *is weakly expressed in maize suspension cultured cells. However, the function of *ZmNIP2;1 *remains to be further characterized precisely. In addition, the expression of rice *OsNIP2;1 *can be regulated by abiotic stresses. For instance, OsNIP2;1 expression is down-regulated during periods of dehydration and in response to the presence of ABA [19], but is induced by salt stress [20].

Homology modeling suggests that NIP proteins share a common general three-dimensional structure [7]. The ar/R filter of *Cicer arietinum *CaNod26 and *Cucurbita pepo *CpNod26 (defined as CaNIP2;1 and CpNIP2;1 respectively in this study) consists of Gly (H2), Ser (H5), Gly (LE1) and Arg (LE2) [7], identical to those NIP III proteins reported in grass species [6,7], and suggests that they also function in silicon uptake and/or translocation [7]. However, for NIP III proteins in dicot species there is no any experimental evidence for the function in silicon uptake and/or translocation, although previous research revealed that the *C. pepo *CpNIP2;1, which is prominent in leaf veins, could catalyze the transmembrane flow of urea and water, but not glycerol [21]. Therefore, it is intriguing to further investigate whether dicot *NIP *III genes can transport larger solutes like silicic acid. Here we demonstrated shifts of selective constraint and positive selection may have been involved in the evolution of the NIP III proteins, which may correspond to functional differences observed between dicot and monocot *NIP *III genes.

## Results and discussion

### Phylogenetic analysis of the NIP III subgroup proteins

The protein and CDS sequences of approximately 20 plant genomes, as well as the cDNA and genomic DNA sequences of six other plant species (*Eragrostis tef*, *Festuca pratensis*, *Elaeis oleifera*, *Musa acuminate*, *Zingiber officinale*, *Curcuma longa*) that belong to Poaceae, Arecaceae, Musaceae, and Zingiberaceae, respectively, were searched. We found that there is at least one *NIP2 *gene presenting in all examined plant species except for two *Arabidopsis *spp. (Additional file 1). In monocotyledonous plants, there are two copies of *NIP2 *encoded within each grass species, except for maize, where three NIP III proteins were characterized [22]; while only one *NIP2 *gene (or fragment) was identified in *Elaeis oleifera*, *Musa acuminate*, *Zingiber officinale *and *Curcuma longa*. However, in the most tested dicotyledonous plants, besides *G. max *and cucumber, only a single copy of *NIP2 *gene was identified. In addition, a homologous NIP2-like protein was found in the licophyte *Selaginella moellendorffii *and the gymnosperm species *Pinus taeda *and *Picea sitchensis*. These results imply that *NIP2 *genes are relatively important for grasses.

**Figure 1:**
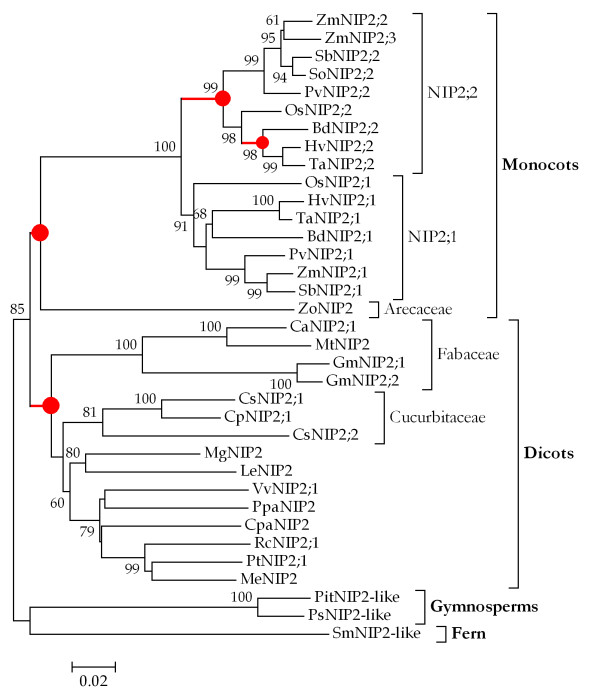
**Phylogenetic tree of *NIP *III subgroup genes in plants**. The tree was reconstructed using the Minimum Evolution (ME) method implemented in MEGA 4.0. The *number *beside the branches represents bootstrap values ≥60% based on 1000 resamplings. Branches with rates of numbers of nonsynonymous and synonymous substitutions >1, are indicated by red thick lines. To identify the species of origin for each *NIP2 *gene, a species acronym is included before the gene name: Bd, *Brachypodium distachyon*; Ca, *Cicer arietinum*; Cp, *Cucurbita pepo*; Cpa, *Carica papaya*; Cs, *Cucumis sativus*; Gm, *Glycine max*; Hv, *Hordeum vulgare*; Le, *Lycopersicon esculentum*; Me, *Manihot esculenta*; Mg, *Mimulus guttatus*; Mt, *Medicago truncatula*; Os, *Oryza sativa*; Pit, *Pinus taeda*; Ppa, *Prunus persica*; Ps, *Picea sitchensis*; Pt, *Populus trichocarpa*; Pv, *Panicum virgatum*; Rc, *Ricinus communis*; Sb, *Sorghum bicolor*; Sm, *Selaginella moellendorffii*; So, *Saccharum officinarum*; Ta, *Triticum aestivum*; Vv, *Vitis vinifera*; Zm, *Zea mays*; Zo, *Zingiber officinale*.

In total, 32 *NIP *III subgroup sequences were used to reconstruct the phylogenetic trees, where the *NIP2-like *homologs in *S. moellendorffii*, *P. taeda *and *P. sitchensis *were used as outgroup sequences to root the trees. It was observed that the ME tree and NJ tree showed the similar topology (Figure 1 and Additional file 2), where two distinct clades were clearly presented: the monocot- and dicot-specific clades (Figure 1), which indicated that the earliest proliferation of *NIP2 *genes in angiosperms occurred after the monocot-dicot split approximately 200 million years ago (Mya) [23].

In the monocot-specific clade, two subclades, based on the support of bootstrap values (91 and 99), were further presented: the NIP2;1 and NIP2;2 subclades (Figure 1). Within each subclade, the orthologous *NIP2 *genes derived from rice, maize, sorghum, barley, wheat, switchgrass, and *B. distachyon *were tightly clustered together, a result suggesting that the proliferation and diversification of *NIP2s *in Poaceae occurred before the divergence of grasses. Since previous studies revealed that the modern grasses diverged from a common grass ancestor [24], and a whole genome duplication (WGD) event was estimated to have occurred about 70 Mya [25,26], we reason that the duplication of *NIP2 *genes in grasses may result from the corresponding WGD. To validate this, we analyzed the syntenic relationship between the chromosomes where the two *NIP2 *genes are located. We observed that three gene pairs including *SbNIP2;1*/*SbNIP2;2*, *BdNIP2;1*/*BdNIP2;2 *(see Additional file 3) and *OsNIP2;1*/*OsNIP2;2 *are located into chromosomal regions that were supposed to have undergone large-scale segmental duplications [1,27]. In maize, *ZmNIP2;2 *and *ZmNIP2;3*, sharing an identity of 93.3% and 91.9% at the protein and DNA sequence level respectively, are closely phylogenetic related (Figure 1), although they are located in different chromosomes (6 and 9). Therefore, the production of *ZmNIP2;2 *and *ZmNIP2;3 *was probably through the recent WGD event occurred in maize [26,28] after its divergence with sorghum from a common ancestor ~12 Mya [29].

In dicotyledonous plants, we found no evidence for the presence of *NIP *III genes in *Arabidopsis thaliana *and *Arabidopsis lyrata*. However, a NIP III protein CpaNIP2;1 was identified in *Carica papaya*, a species sharing a common ancestor with *A. thaliana *~72 Mya [23]. Thus, it is parsimonious to infer that the *NIP *III gene in *Arabidopsis *spp. were probably lost after their speciation from *C. papaya*. In addition, we observed that the four *NIP *III genes derived from three legumes (*C. arietinum*, *M. truncatula*, and *G. max*) were clustered together and presented as a Fabaceae-specific clade (Figure 1). The D_4DTv _value of soybean *GmNIP2 *genes is very small (0.087), indicative of their recent duplication. Since the soybean (*G. max*) genome has undergone two rounds of large-scale genome and/or segmental duplication at about 14 and 42 Mya, respectively [30,31], it is likely that the two *GmNIP2s *were produced via the recent instead of the ancient large-scale duplication event. Similarly, the three Cucurbitaceae *NIP2 *genes constituted a lineage-specific clade, where two *NIP2s *(*CsNIP2;1 *and *CsNIP2;2*) were derived from *Cucumis sativus*. From the phylogenteic tree (Figure 1), it can be inferred that the duplication of *CsNIP2;1 *and *CsNIP2;2 *possibly predated the speciation time of cucumber and zucchini. Analysis of the cucumber genome sequence successfully identified an ancient WGD, but did not reveal recent duplications [32]. Using a global clock model implemented in PAML [33], we estimated the divergence time of the two *CsNIP2s *at ~67.7 Mya; their D_4DTv _value is much larger (0.441) (Table 1), suggestive of their ancient evolutionary past. Interestingly, only one *NIP2 *gene (*PtNIP2;1*) was identified in the poplar genome, although it has experienced two rounds of WGD [34].

**Table 1:** 4DTv distance (D_4DTv_) between paralogous *NIP2 *genes in monocot and dicot plants

Species	Gene pair	**D**_**4DTv **_**value**	Species	Gene pair	**D**_**4DTv **_**value**
*O. sativa*	OsNIP2;1/OsNIP2;2	0.319	*Z. mays*	ZmNIP2;1/ZmNIP2;2	0.260
*T. aestivum*	TaNIP2;1/TaNIP2;2	0.309		ZmNIP2;1/ZmNIP2;3	0.313
*H. vulgare*	HvNIP2;1/HvNIP2;2	0.403		ZmNIP2;2/ZmNIP2;3	0.128
*B. distachyon*	BdNIP2;1/BdNIP2;2	0.366	*G. max*	GmNIP2;1/GmNIP2;2	0.087
*P. virgatum*	PvNIP2;1/PvNIP2;2	0.299	*C. sativus*	CsNIP2;1/CsNIP2;2	0.441
*S. bicolor*	SbNIP2;1/SbNIP2;2	0.260			

### Sequence characteristic analysis

In grasses, the orthologous NIP III subgroup proteins are significantly homologous to each other (Figure 1). The average identity of sequences for the NIP2;1 and NIP2;2 subclade proteins is 85.9% and 87.5% respectively, indicating that the NIP2 orthologs within each subclade can perform the same or similar functions. In agreement with this postulation, barley HvNIP2;1 (HvLsi1), like rice OsNIP2;1 (OsLsi1), was demonstrated to function in mediating the influx of silicic acid in roots [9,10]. In addition, we calculated the D_4DTv _values for the paralogous genes in grasses, and found that with one exception (*ZmNIP2;2*/*ZmNIP2;3 *with a D_4DTv _value 0.128), other paralogous gene pairs are highly diverged, having a D_4DTv _value not less than 0.26 (Table 1). Therefore, the sequence variation should be partly responsible for their functional diversification, as indicated by the functional studies of OsNIP2;1 and OsNIP2;2 [10,11,13].

In contrast to the high similarity in grasses, large sequence variation was observed among dicot NIP2s that share an average identity of 61.6%. In addition, we found that the overall average identity between monocot and dicot NIP2s is decreased up to 60.4%. Nonetheless, both monocot and dicot *NIP2s *show similar gene structures, possessing five exons and four introns (Additional file 4). Although the introns vary extensively in length, three exons (from 2 to 4) are remarkablely conserved in tested plants (see Additional file 4), suggesting that strong functional constraints should impose on the corresponding exonic regions. In the two soybean *GmNIP2s*, both the exons and introns are slightly different in length, supporting of their recent expansion. However, substantial variation is found in the intron lengths, especially in the second and fourth introns for the *ZmNIP2;2*/*ZmNIP2;3 *gene pair, implying that their diversification may be ongoing. The similar cases were also observed in cucumber (*CsNIP2;1*/*CsNIP2;2*) and other paralogous gene pairs in grasses.

**Figure 2:**
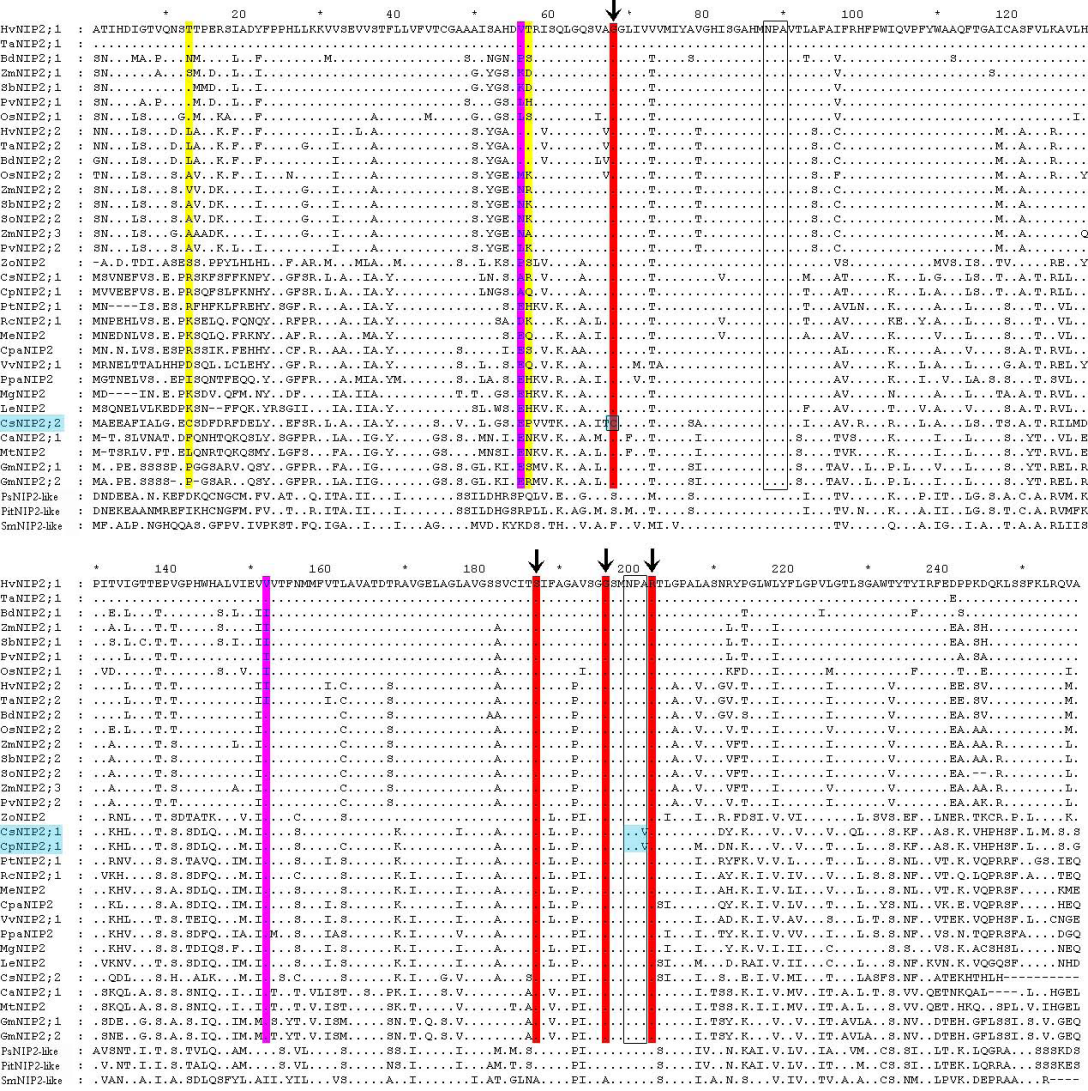
**Multiple sequence alignment of plant NIP III protein sequences**. In the manually modified alignment, the residues are displayed in the "Difference Mode" with the "Diff/Consensus Line" style. Dots indicate conserved residues with the first protein HvNIP2;1, and "-" indicates gaps on the alignment. The dual NPA motifs are boxed. The four residues making up the ar/R filter are designated with arrows and highlighted in red. Positively selected sites are shadowed in yellow. The critical amino acid sites (CAASs) responsible for functional divergence (*Q*_k _> 0.9) are shaded in purple, and highlighted in blue (Type-I).

As reported, the dual conserved NPA motifs and ar/R filter play determinant roles for the selectivity of aquaporin proteins [35]. The ar/R filter is located in the narrowest region on the extra-membrane mouth of the pore, which is approximately 8Å above the NPA region [7]. We observed that the four residues making up the ar/R filter are G, S, G, and R in tested proteins, which is consistent with the typical feature of NIP III proteins, except for CsNIP2;2, where the first residue in the H2 is C instead of G (Figure 2; [1]). We cloned and re-sequenced the *CsNIP2;2 *gene, and confirmed the replacement of G with C. As the composition of ar/R filter defines the pore size, pore hydrophobicity and hydrogen bonding between pore and substrate [36], the substitution of the tiny Gly (G) residue with the bulkier Cys (C) may give rise to a much narrower aperture than other NIP III proteins (Additional file 5). Therefore, functional analysis is urgently needed to further investigate why the cucumber genome has evolved to two NIP III proteins, but one of them has possibly lost its ability for permeating larger solutes, such as silicic acid. As for the NPA motifs, we found that the first NPA motif is markedly conserved, whereas the second one is slightly variant, with the substitution by NPV in CsNIP2;1 and CpNIP2;1 (Figure 2). NIP proteins often have unorthodox NPA motifs [1], which are different from other MIPs. Furthermore, mutations in the NPA motifs do not change the selectivity of NIP proteins [37], it thus seems that the NPA motif is not a crucial factor in determining the substrate selectivity for NIPs [6,8]. However, whether the NPA motifs play critical roles in NIP III proteins remains to be further examined, since the NPA motifs are highly conserved in NIP III proteins, only with the exceptions of CsNIP2;1 and CpNIP2;1.

### Positive selection in the *NIP2 *gene sequences

The CODEML program implemented in the PAML v4.4 software package [33] was utilized to test the hypothesis of positive selection in the *NIP *III subgroup genes. The estimation of positive selection was based on the tree topology shown in Figure 1, where only full length sequences were included into analysis. To test whether there are variable ω ratios at amino acid sites, two pairs of models (M0/M3, and M7/M8) were selected and compared. In the model M0, a single ω is assumed for all sites in the alignment. Under this model, the estimated ω value is 0.197 for *NIP2s *with the log-likelihood score ℓ = -13752.4. Compared to other models, M0 shows a worse fit for the data because of its much lower log-likelihood value than all other models (Table 2), and thereby ruling out the possibility that all sites in the alignment have the same ω ratio. In contrast, the selection models (M3 and M8) fit the data significantly better than those that do not permit positive selection (M0 and M7) (Table 2), indicating that the *NIP2 *genes should be under adaptive evolution where some sites might undergo amino acid substitutions with high rate.

**Table 2:** Results of positive selection analysis using a variety of codon substitution models

Model	Omega distribution	ln*L*	2Δℓ	**Positively selected sites**^**a,b**^
M0 (one ratio)	ω = 0.197	-13752.4		None

M3 (discrete)	0.97% sites: ω = 1.85; 99.03% sites: 0.01<ω < 0.80	-13171.3	1162.2 (M3 *vs *M0) *p *< < 0.01	28T*, 77S*

M7 (β)	ω = 0.243	-13181.8		Not allowed

M8 (β+ω > 1)	1.72% sites: ω = 1.54; 98.28% sites: 0.0004<ω < 0.76	-13176.7	10.2 (M8 *vs *M7) *p *< 0.01	28T*, 30P, 38A, 77S*, 245M

*Note*: ^a^, Codon sites under positive selection that are estimated using the program CODEML implemented in the PAML package v4.4. Asterisk (*) denotes posterior probability > 0.95.

^b^, Codon (amino acid) positions presented above are based on the rice *OsNIP2;1 *gene.

The comparison of M3 *vs *M0 reveals that ω is not uniformly distributed along the *NIP2 *coding DNA sequences, and about 0.97% codon sites may be under the influence of positive selection (ω = 1.85). Similarly, compared with M7 model, the M8 model suggests that ~1.72% of codons fall in a category with estimated ω value 1.54, a result indicative of strong positive selection.

**Figure 3:**
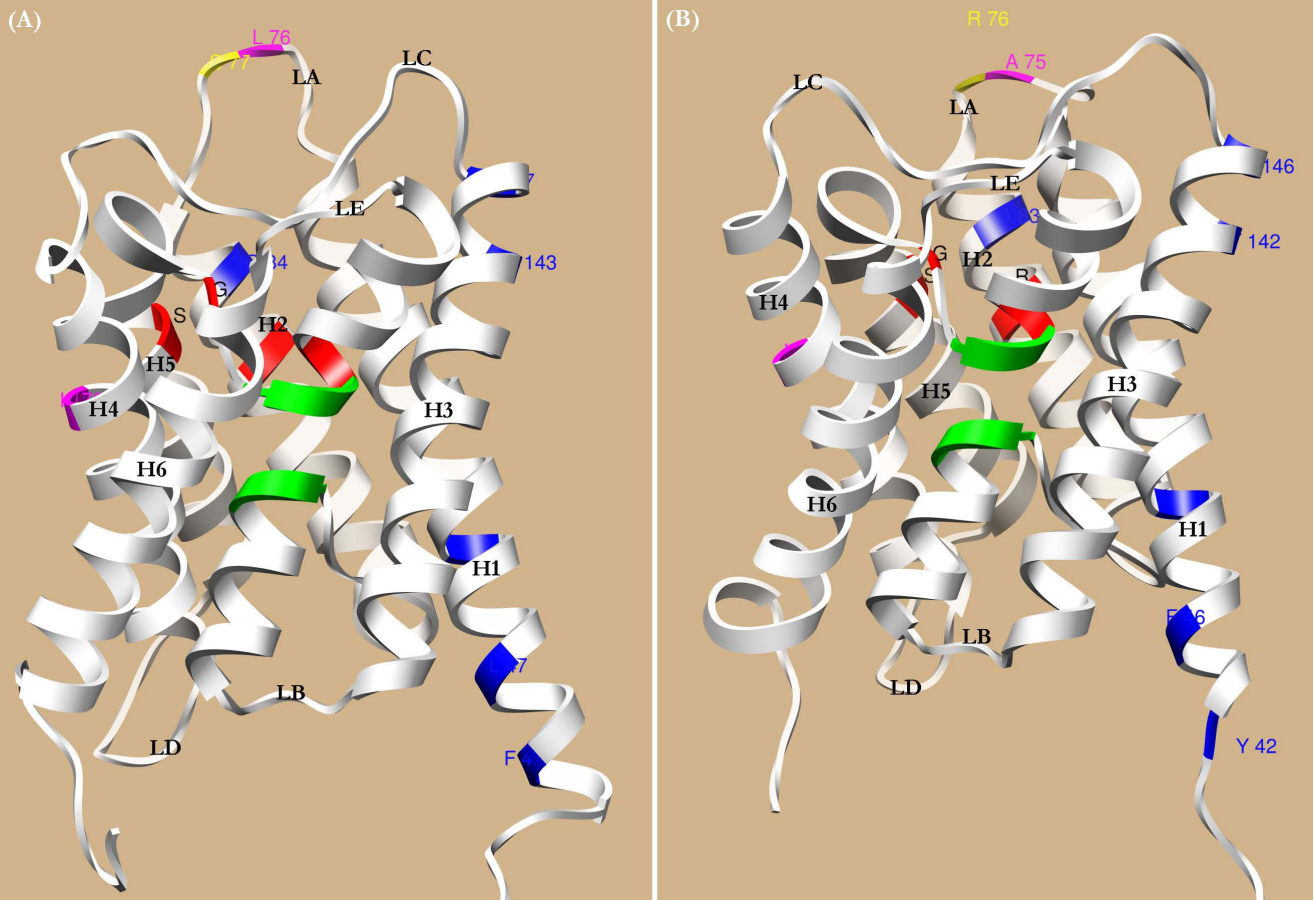
**Rice OsNIP2;1 (A) and cucumber CsNIP2;1 (B) protein structures**. The corresponding structures are predicted using HHpred and presented in the form of edged Ribbon, where the six helixes and five loops are indicated. The NPA motifs and ar/R selectivity filter are colored in green and red respectively. Positively selected sites and Type-I functional divergence related amino acid sites are highlighted in yellow and purple respectively. The predicted SDPs responsible for the functional specificity between dicot and monocot NIP III proteins are shadowed in blue.

Based on the Bayesian posterior probabilities, two and 5 codon site candidates for positive selection were identified for the M3 and M8 models respectively, of which the posterior probabilities of sites 28 and 77 are >0.95 in both analyses (Table 2). In the M8 model, the posterior probabilities of three site candidates (30P, 38A, and 245M) are all less than 0.7, and thereby excluded from further analysis. Furthermore, we observed that sites 28 and 77 are located in exons 1 and 2, respectively. After projecting the two sites onto the simulated three-dimensional structures, we clearly observed that the first site (28T) is located in the N-terminus, while the other site (77S) in the periplasmic LA (loop A) (Figures 2 and 3).

Using a variety of codon site models, we demonstrated the influence of positive selection in the evolution of *NIP *III subgroup genes (Table 2). Compared with the helix regions, the N- and C-terminus are highly divergent in NIP2 proteins (see Additional file 6). Nevertheless, it was found that plant aquaporins were regulated by phosphorylation within the cytosolic termini and loop regions [35,38]. We used the NetPhos2.0 Server [39] to predict any possible serine and threonine phosphorylation sites in NIP2 proteins, and indeed found many predicted candidates in the corresponding regions (Additional file 7). These results suggest that the N-terminus and loop regions are functionally important, although they are not conserved as much as the helix regions. The site 77 is located in the loop between helix H1 and H2 (LA). In rice OsNIP2;1, the residue at the corresponding site is Ser (S), which was predicted to be a Serine phosphorylation site with high possibility (score = 0.89; Additional file 7); whereas in other NIP2s, the site is occupied by variant amino acids with different physiochemical properties (Figure 2). Therefore, positive selection in these regions may act as one major determinant in driving the functional divergence of NIP2s.

To test whether variable ω ratios are present among lineages, we performed the likelihood ratio test (LRT) to compare the two extreme models: the one-ratio model that assumes a unique rate ratio for all branches, and the free-ratio model that assumes an independent ω ratio for each branch [33]. The log-likelihood value under the one-ratio model is -11945.3, while the value is -11796.9 for the free-ratio model. Twice the log likelihood differences, 2Δℓ = 296.8, is strongly statistically significant (*p *< 0.01), revealing a heterogeneous selective pressures among lineages. We further observed that some branches of the *NIP *III gene phylogeny include several internal branches having ω >1 (Figure 1), showing strong evidence for adaptive evolution.

In a gene family, the fate of new genes produced by duplication would either evolve a new function under positive selection, or be lost during evolution [40]. As reported, plants have evolved more NIP proteins with multi-functions [1,16,22]. We revealed that positive selection was involved in the functional diversification of *NIP *subfamily genes [1], as well as in the evolution of *NIP *III subgroup genes (Figures 1 and 2, and Table 2). The grasses encoded two or three *NIP2 *genes, where the *NIP2;2 *gene has evolved new functions differentiating from that of *NIP2;1s*, which may represent an evolutionary advantage for grasses to uptake and translocate silicon efficiently.

### Functional divergence analysis of NIP2 proteins

The Gu (1999; 2006) methods [41,42] implemented in DIVERGE2 [42] were used to evaluate Type-I (shifted evolutionary rate) and Type-II (altered amino acid physiochemical property) functional divergence between gene clusters of interests in the NIP III subgroup. The advantage of these methods is that they use amino acid sequences, and thereby is not sensitive to saturation of synonymous sites [41,42].

Based on the distinct expression patterns and functions of OsNIP2;1 and OsNIP2;2 [10,11,13], we supposed that functional divergence in NIP2s should have occurred after gene duplication in grasses. Unexpectedly, we found no statistical evidence for Type-I functional divergence between NIP2;1 s and NIP2;2s in grasses, because the coefficient of Type-I functional divergence was insignificant (*θ*_I _= 0.086 ± 0.145; LRT = 0.44; *p *> 0.05). However, when the posterior probability (Q*_k_*) of divergence was determined for each site by DIVERGE2 [42], 7 sites with high probability (0.9 > Q*_k _*> 0.8) were identified to be Type-II functional divergence related (Additional file 8), indicative of a radical shift of amino acid properties [42]. It appears that site-specific changes of amino acid physiochemical properties may act as one of the major evolutionary powers in driving the functional divergence of NIP2 proteins after their duplication in grasses. Of the seven sites identified, 5 of them are located in helixes and two in loop regions (LB and LE) (Additional file 8). Further, we observed that although the three sites located in helix H2 (V/T), H5 (A/P), and LE (G/A) are physically close to the four residues of the ar/R filter (G, S, G, and R) in the amino acid sequences, only G/A in LE is predicted to spatially interact with R in LE2. In addition, we found that the two residues in LB (A/S) and LE (G/A) are not only physically adjacent to the dual NPAs, but spatially interact with the third residue (A) in the NPA motifs. LB and LE are functional loops that participate in the formation of the aqueous pore [35], implying that the A/S and G/A residues should be structurally important. Moreover, a contact between LE (G/A) and helix H3 (S/A) was also observed. Therefore, the radical change of physiochemical property of the 7 sites may be the primary contributor to the functional divergence of NIP2s in grasses.

We further employed the DIVERGE2 to assess the coefficients of Type-I and Type-II functional divergence (*θ*_I _and *θ*_II_) between monocotyledonous and dicotyledonous NIP2s. We found that the null hypothesis (no functional divergence) can be strongly rejected, because the *θ*_I _value is statistically significant (*θ*_I _= 0.145 ± 0.041; LRT = 12.6; *p *< 0.01). This indicates that shifted selective constraints must strongly operate on some amino acid sites in monocot and dicot NIP2s, and thereby leading to a lineage-specific functional evolution after their divergence from an ancient common ancestor. Furthermore, two critical amino acid sites (CAASs) were predicted to be highly Type-I functional divergence related (Q*_k _*> 0.9), where one site is located in LA, and the other in the helix H4 (Figures 2 and 3). The CAAS in LA (76L) is proximal to the identified positively selected site 77 S. Using InterMap3 D [43], the two sites are predicted to spatially contact with each other, and have probably coevolved during evolution. In dicot NIP2s, the CAAS in H4 is invariant Val (V), whereas it is substituted with Ile (I) in some monocot NIP2s. Nevertheless, no obviously statistical evidence for the Type-II functional divergence was found (*θ*_II _= -0.030 ± 0.096; *p *> 0.05). Together, these observations indicated that amino acid site-specific shifts of evolutionary rate and changes of amino acid property should not uniformly act on the NIP2s after the split of monocots and dicots.

### Determination of functional specificity positions among orthologous NIP2 proteins

Many protein families, such as the aquaporin superfamily, contain homologous proteins that have a common biological function but with different specific substrates and interactive molecules [44]. It is thus necessary to identify residues that are significantly responsible for the functional specificity, which may be useful in biological studies. To this end, we used the SDPpred [44] server to predict any possible Specificity Determining Positions (SDPs) that may determine the functional specificity of orthologous NIP2 proteins after the monocot-dicot split. As shown in Table 3 and Figure 3, 5 SDPs were identified to be highly relevant to functional specificity. Besides, we also observed that 3 out of the five SDPs are located in the N-terminus, and the other two SDPs lie on helix H3 (Figure 3 and Additional file 6).

**Table 3:** Specificity determining positions (SDPs) in the monocot and dicot lineage-specific NIP2 proteins

No.	Alignment position	Position in			Mutual information	Bernoulli estimator (B-cutoff)
				
		HvNIP2;1	CsNIP2;1	ZoNIP2		
1	104	***84Q***	***83A***	***72A***	0.67	0.000
2	163	143V	142T	131V	0.67	-2.977
3	73	53S	52A	41S	0.66	-6.402
4	167	147V	146L	135V	0.66	-10.312
5	63	43F	42Y	31L	0.66	-11.135
6	67	47L	46F	35L	0.66	-13.372

*Note*: "Alignment position" designates the amino acid position in the multiple sequence alignment presented in Additional file 6. The amino acid site 84Q that is predicted to be a possible SDP is shown in bold and italic.

In addition, we noticed that when using OsNIP2;1 as the reference sequence, the residue at the site 84 was invariant Gln (Q) in monocots including *Musa acuminate *and *Elaeis oleifera*, except for the *Zingiber officinale *ZoNIP2, where the corresponding site was occupied by Ala (72A) (Additional files 1 and 6). Whether the substitution of Q with A in ZoNIP2 is sequencing or assembling error is unknown. However, if the residue 72A in ZoNIP2 was replaced by G, this site was also predicted to be a SDP with high confidence (*p *value = 0.000; Table 3). Moreover, it was found that the residue 84Q lies on the channel side (Additional file 9), and exhibits an interaction with the first residue of the ar/R filter in H2. This suggests that the site 84Q may be involved in the formation of a functional pore, although the ar/R residues are identical in rice OsNIP2;1 and cucumber CsNIP2;1 (Figure 2 and Additional file 5). If it was true, the *Z. officinale ZoNIP2 *gene, like dicots, may perform a dissimilar function from other monocots.

Notably, we found that the predicted SDPs for the NIP III subgroup are mainly distributed in two spatial regions. As discussed above, the site 84Q was postulated to be involved in the formation of the channel (Additional file 9). By contrast, most of other SDPs lie on the surface of the protein and likely participate in establishing the tetrameric structure.

## Conclusions

The current NIP III subgroup proteins were originated and diverged from an ancient common ancestor that emerged before the divergence of monocots and dicots. Subsequently, monocot- and dicot-specific expansion of *NIP2 *genes occurred. Plant *NIP2s *show relatively conserved gene structures, each containing five exons and four introns. With only one exception (CsNIP2;2), the ar/R filter of NIP2 proteins consists of G, S, G, and R.

All tested grasses encoded at least two NIP2 proteins in their genomes. These paralogous NIP2s were probably produced via the whole genome duplication (WGD) or segmental chromosomal duplication event occurred in the common ancestor of modern grasses. The proliferation and diversification of NIP2s in grasses may be, at least partially, responsible for their highly efficient influx of silicon in roots and then transporting them out of xylem. By contrast, most of tested dicot plants have only one *NIP2 *gene. Due to the small constriction size of the pore, CsNIP2;2 might be unable to transport silicon, although the cucumber genome encoded two *NIP2 *genes. In particular, *Arabidopsis *spp. should have lost their *NIP *III subgroup genes during evolution.

The *NIP *III subgroup genes have experienced strong positive selection and diverged in function between monocots and dicots. Several SDPs were identified to be responsible for the determination of functional specificity of monocot and dicot NIP2 proteins. These findings provide deeper insights into understanding the evolutionary mechanisms of NIP III subgroup proteins and their functional diversification.

## Methods

### Sequence data

The rice (OsNIP2;1 and OsNIP2;2), sorghum (SbNIP2;1 and SbNIP2;2), maize (ZmNIP2;1, ZmNIP2;2 and ZmNIP2;3), barley HvLsi1 (HvNIP2;1), *C. arietinum *CaNIP2;1 and *C. pepo *CpNIP2;1 proteins that belong to the NIP III subgroup were collected according to the published literatures [1,7,9-11,22]. The *Ricinus communis *RcNIP2;1 (AN: EEF27965) was identified on the basis of a BLASTP search against the nr database with *e value *0.01 in National Center for Biotechnology Information (NCBI). These proteins were used as query to search against the amino acid and DNA sequences of the *Brachypodium distachyon*, *Triticum aestivum*, *Hordeum vulgare*, *Vitis vinifera*, *Carica papaya*, *Medicago truncatula*, *Glycine max*, *Lycopersicon esculentum*, *Populus trichocarpa*, *Cucumis sativus*, *Panicum virgatum*, *Mimulus guttatus*, *Prunus persica*, and *Manihot esculenta *genomes using BLASTP and TBLASTN programs, respectively. In addition, an exhaustive search against the cDNA and/or genomic DNA sequences of the genomes *Eragrostis tef*, *Festuca pratensis*, *Elaeis oleifera*, *Musa acuminate*, *Zingiber officinale*, *Curcuma longa*, *Picea sitchensis*, and *Selaginella moellendorffii *were performed also using BLASTN and TBLASTN programs, respectively. Programs InterProScan [45] and ConPred II [46] were utilized to detect conserved domains and predict the putative transmembrane regions (TMs), respectively. FgeneSH http://linux1.softberry.com/ was employed to predict the gene structures of candidates identified.

### Multiple sequence alignment and phylogenetic tree reconstruction

The NIP III subgroup protein sequences were aligned using the program L-INS-i implemented in MAFFT v6.6 [47], with the parameters: Scoring matrix for amino acid sequences, BLOSUM62; Gap opening penalty, 2.0; and Gap extension penalty, 0.2. The resulting protein alignment was subsequently employed to generate the codon-alignment of corresponding coding DNA sequences using a custom PERL script. In the codon-based alignment, the codon sites at which most sequences have data except for one or two sequences were kept while sites at which all sequences except for one or two have alignment gaps were removed. The phylogenetic trees were reconstructed with MEGA v4.0 [48] using the Minimum Evolution (ME) and Neighbor-joining (NJ) methods with the parameters of pairwise deletion of gaps/missing data and the *p*-distance substitution model where only the transversions were taken into account. The reliability of interior branches was assessed with 1000 bootstrap resamplings. Phylogenetic trees were displayed using MEGA v4.0 [48].

### Tests of positive selection

We employed the CODEML program implemented in the PAML v4.4 software package [33] to test the hypothesis of positive selection in the *NIP *III subgroup genes. In the analysis, two pairs of models were contrasted to test for heterogeneous selective pressures at codon sites. First, models M0 (one ratio) and M3 (discrete) were compared, using a test for heterogeneity between codon sites in the *d*_N_/*d*_S _ratio value, ω. The second comparison was M7 (beta) *vs *M8 (beta+ω >1); this is the most stringent test of positive selection [49]. M0 and M7 belong to null models that do not allow for any codons with ω >1. In such comparisons, positive selection is indicated if the models that allow for selection (M3 and M8) are significantly better than the null model (no selection) in the likelihood ratio test (LRT). When the LRT suggests positive selection, the Bayes empirical Bayes (BEB) method was used to calculate the posterior probabilities that each codon is from the site class of positive selection under models M3 and M8 [50].

To test for heterogeneous selective pressures among lineages [33], models of variable ω ratios among lineages were fitted by maximum likelihood (ML) to the *NIP *III subgroup sequence alignment. The ratio of nonsynonymous-to-synonymous for each branch under two models (one-ratio and free-ratio for branches) was calculated, and the two models were compared using the LRT test to see whether the ω ratios are different among lineages. Accordingly, positive selection is indicated if the free-ratio model that allows for selection is significantly better than the one-ratio model (no selection) in the LRT analysis.

### Estimation of functional divergence

The software DIVERGE2 [42] was employed to detect functional divergence between clusters of interests in the plant *NIP *III subgroup. The coefficients of Type-I and Type-II functional divergence (*θ*_I _and *θ*_II_) between any two interesting clusters were calculated. If *θ*_I _or *θ*_II _is significantly >0, it means site-specific altered selective constraints or a radical shift of amino acid physiochemical property after gene duplication and/or speciation [41,42]. Moreover, a site-specific profile based on the posterior probability (*Q_k_*) was used to predict critical amino acid residues that were responsible for functional divergence. In this analysis, large *Q_k _*indicates a high possibility that the functional constraint (or the evolutionary rate) and/or the radical change of amino acid property of a site is different between two clusters [41,42].

### Analysis of specificity determining positions

The SDPpred [44] was utilized to identify the specificity determining positions (SDPs) that determined differences in the functional specificity of homologous proteins. Based on a given multiple alignment, SDPpred computes *Z-*score for each alignment column. The higher *Z*-score, the more likely the position is a true specificity determinant. The Bernoulli estimator was incorporated into SDPpred to automatically produce a recognition cutoff (B-cutoff) to evaluate the significance of the *Z*-scores in order to assess whether the observed *Z*-score is sufficiently high to indicate an SDP. Positions scoring higher than this cutoff are predicted to determine the specificity.

### Homology molecular modeling

The homology models of NIP III subgroup proteins were generated with HHpred [51] using the method of HMM-HMM comparison of the queried protein and the templates deposited in the PDB database. The generated models were prepared and viewed with UCSF Chimera [52], and the critical amino acid residues identified were mapped onto the corresponding structures accordingly.

### 4DTv calculation

4DTv distance (D_4DTv_) that stands for fourfold synonymous third-codon transversion was calculated to assess the genetic distances between paralogous pairs. In such analysis, the paralogous proteins for each species were pairwise aligned using MAFFT v6.6 [47], and subsequently the corresponding codon-alignment was created according to the resulting protein alignment using a custom PERL script. Based on these alignments, we firstly identified the conserved fourfold degenerate amino acids. And then the corresponding codons were extracted from the codon-alignment and used to calculate the 4DTv distance between each aligning pair. D_4DTv _ranges from 0 for recently duplicated peptides, to ~0.5 for paralogs with an ancient evolutionary past.

## Authors' contributions

QL and ZZ conceived and designed the experiments. QL performed the experiments, analyzed the data and wrote the paper. ZZ cloned the cucumber *CsNIP2;1 *and *CsNIP2;2 *genes, participated in the discussion and revision of the earlier manuscript. All authors read and approved the final manuscript.

## Supplementary Material

Additional file 1 **Multiple sequence alignment and NJ phylogenetic tree of *NIP *subfamily genes in plants**. (A) The amino acid sequences of plant NIP subfamily proteins were aligned using the program L-INS-i implemented in MAFFT v6.6. In the alignment, the residues are displayed in the "Difference Mode" with the "Diff/Consensus Line" style. Dots indicate conserved residues with the first protein HvNIP2;1, and "-" indicates gaps on the alignment. The dual conserved NPA motifs are boxed. In NIP III, the four residues making up the ar/R filter are highlighted in red and blue for the angiosperms and other plants respectively. (B) The phylogenetic tree was reconstructed using the Neighbor-Joining (NJ) method implemented in MEGA 4.0. The *number *beside the branches represents bootstrap values ≥ 60% based on 1000 resamplings. Click here for file

Additional file 2 **Neighbor-Joining (NJ) tree of *NIP *III subgroup genes in plants**. The *number *beside the branches represents bootstrap values ≥ 60% based on 1000 resamplings. Click here for file

Additional file 3 **Distribution of *NIP2 *genes and segmental duplication events contributed to the evolution of *NIP *III subgroup in sorghum (A) and *Brachypodium *(B)**. The gene coordination files and predicted amino acid sequences for the sorghum and *Brachypodium *genomes were downloaded from Phytozome http://www.phytozome.net. The predicted amino acid sequences were separated into different chromosomes according to the annotation information derived from the gene coordination files. The amino acid sequences within each chromosome were searched against each other using BLASTP. The hits with an *e value *less than 1e-5 were used as input for the Blast Synteny Toolkit (version 06132003) that was downloaded from TIGR, with the default parameters to generate the corresponding syntenic figures. Click here for file

Additional file 4 **The exon/intron lengths and gene structure of *NIP2 *genes in monocot and dicot plants**. Click here for file

Additional file 5 **Spatial organization of residues forming the ar/R filter of the proximal part (located at the extracellular face) of the water channel**. (A) Cucumber CsNIP2;1; (B) Cucumber CsNIP2;2; (C) Zucchini CpNIP2;1; (D) Barley HvNIP2;1; (E) Rice OsNIP2;1. The ar/R selectivity filter of NIP III proteins in dicot and monocot plants is composed of G, S, G, and R, with only one exception (CsNIP2;2), where the first residue in H2 was replaced by the bulkier Cys (C). Click here for file

Additional file 6 **Illustration of specificity determining positions (SDPs) in monocot and dicot plants**. In the multiple-alignment of full-length NIP III protein sequences, the residues are displayed in the "Difference Mode" with the "Diff/Consensus Line" style. Dots indicate conserved residues with the first protein HvNIP2;1, and "-" indicates gaps on the alignment. The possible Specificity Determining Positions (SDPs) that might determine the functional specificity of orthologous NIP2 proteins after the monocot-dicot split are shaded in green. The dual NPA motifs are boxed. The four residues making up the ar/R filter are designated with arrows and highlighted in red. Click here for file

Additional file 7 **The predicted serine and threonine phosphorylation sites in plant NIP III proteins**. Click here for file

Additional file 8 **Significantly Type-II functional divergence related amino acid sites in grasses**. The six putative transmembrane regions (TMs) are shadowed in black. The 7 amino acid candidates identified responsible for the Type-II functional divergence between NIP2;1 and NIP2;2 proteins are highlighted in red. Click here for file

Additional file 9 **Simulated structures of rice OsNIP2;1 (A-B) and cucumber CsNIP2;1 (C-D)**. Specificity-determining positions (SDPs) are colored in blue. The possible SDP 84Q (OsNIP2;1) or 83A (CsNIP2;1) is circled and designated with arrow. The NPA motifs and ar/R filter are highlighted in green and red respectively. Positively selected sites and significantly Type-I functional divergence related sites are shaded in yellow and purple respectively. Click here for file
