# Supplementary material for: Functional divergence of the NIP III subgroup proteins involved altered selective constraints and positive selection
Source: BMC Plant Biol. 2010 Nov 20;10:256. doi: 10.1186/1471-2229-10-256 (PMC3095335; doi:10.1186/1471-2229-10-256)
Supplement: Additional file 1 — Multiple sequence alignment and NJ phylogenetic tree of NIP subfamily genes in plants. (A) The amino acid sequences of plant NIP subfamily proteins were aligned using the program L-INS-i implemented in MAFFT v6.6. In the alignment, the residues are displayed in the "Difference Mode" with the "Diff/Consensus Line" style. Dots indicate conserved residues with the first protein HvNIP2;1, and "-" indicates gaps on the alignment. The dual conserved NPA motifs are boxed. In NIP III, the four residues making up the ar/R filter are highlighted in red and blue for the angiosperms and other plants respectively. (B) The phylogenetic tree was reconstructed using the Neighbor-Joining (NJ) method implemented in MEGA 4.0. The number beside the branches represents bootstrap values ≥ 60% based on 1000 resamplings. [file 1471-2229-10-256-S1.DOC]

**（A）**


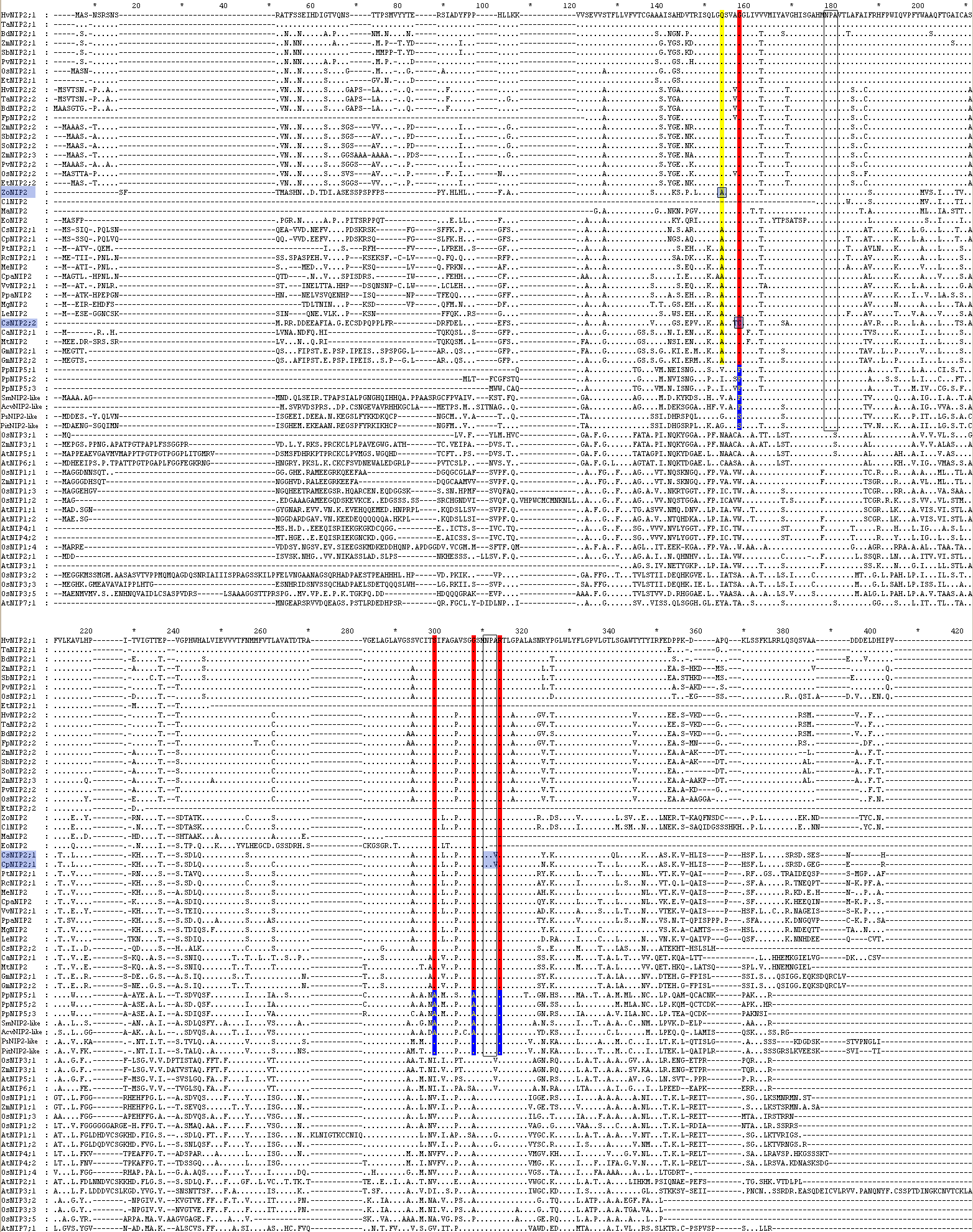


**（B）**

**Additional file 1**

Multiple sequence alignment and NJ phylogenetic tree of *NIP* subfamily genes in plants. (A) The amino acid sequences of plant NIP subfamily proteins were aligned using the program L-INS-i implemented in MAFFT v6.6. In the alignment, the residues are displayed in the “Difference Mode” with the “Diff/Consensus Line” style. Dots indicate conserved residues with the first protein HvNIP2;1, and “−” indicates gaps on the alignment. The dual conserved NPA motifs are boxed. In NIP III, the four residues making up the ar/R filter are highlighted in red and blue for the angiosperms and other plants respectively. (B) The phylogenetic tree was reconstructed using the Neighbor-Joining (NJ) method implemented in MEGA 4.0. The *number* beside the branches represents bootstrap values ≥ 60% based on 1000 resamplings. To identify the species of origin for each *NIP2* gene, a species acronym is included before the gene name: Acv, *Adiantum capillus-veneris*; At, *Arabidopsis thaliana*; Bd, *Brachypodium distachyon*; Ca, *Cicer arietinum*; Cl, *Curcuma longa*; Cp, *Cucurbita pepo*; Cpa, *Carica papaya*; Cs, *Cucumis sativus*; Eo, *Elaeis oleifera*; Et, *Eragrostis tef*; Fp, *Festuca pratensis*; Gm, *Glycine max*; Hv, *Hordeum vulgare*; Le, *Lycopersicon esculentum*; Ma, *Musa acuminate*; Me, *Manihot esculenta*; Mg, *Mimulus guttatus*; Mt, *Medicago truncatula*; Os, *Oryza sativa*; Pit, *Pinus taeda*; Pp, *Physcomitrella patens*; Ppa, *Prunus persica*; Ps, *Picea sitchensis*; Pt, *Populus trichocarpa*; Pv, *Panicum virgatum*; Rc, *Ricinus communis*; Sb, *Sorghum bicolor*; Sm, *Selaginella moellendorffii*; So, *Saccharum officinarum*; Ta, *Triticum aestivum*; Vv, *Vitis vinifera*; Zm, *Zea mays*; Zo, *Zingiber officinale*.
