# Supplementary material for: Functional divergence of the NIP III subgroup proteins involved altered selective constraints and positive selection
Source: BMC Plant Biol. 2010 Nov 20;10:256. doi: 10.1186/1471-2229-10-256 (PMC3095335; doi:10.1186/1471-2229-10-256)
Supplement: Additional file 2 — Neighbor-Joining (NJ) tree of NIP III subgroup genes in plants. The number beside the branches represents bootstrap values ≥ 60% based on 1000 resamplings. [file 1471-2229-10-256-S2.DOC]

**Additional file 2**

Neighbor-Joining (NJ) tree of *NIP* III subgroup genes in plants. The *number* beside the branches represents bootstrap values ≥ 60% based on 1000 resamplings. The species acronym is consistent with those shown in figure 1.
