# Supplementary material for: Functional divergence of the NIP III subgroup proteins involved altered selective constraints and positive selection
Source: BMC Plant Biol. 2010 Nov 20;10:256. doi: 10.1186/1471-2229-10-256 (PMC3095335; doi:10.1186/1471-2229-10-256)
Supplement: Additional file 3 — Distribution of NIP2 genes and segmental duplication events contributed to the evolution of NIP III subgroup in sorghum (A) and Brachypodium (B). The gene coordination files and predicted amino acid sequences for the sorghum and Brachypodium genomes were downloaded from Phytozome http://www.phytozome.net. The predicted amino acid sequences were separated into different chromosomes according to the annotation information derived from the gene coordination files. The amino acid sequences within each chromosome were searched against each other using BLASTP. The hits with an e value less than 1e-5 were used as input for the Blast Synteny Toolkit (version 06132003) that was downloaded from TIGR, with the default parameters to generate the corresponding syntenic figures. [file 1471-2229-10-256-S3.DOC]

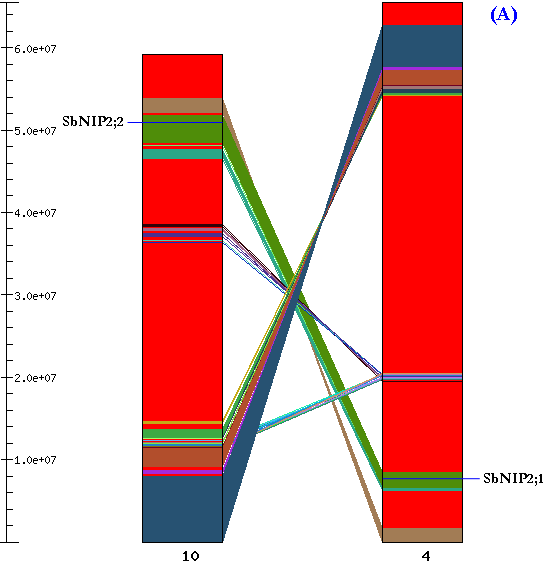

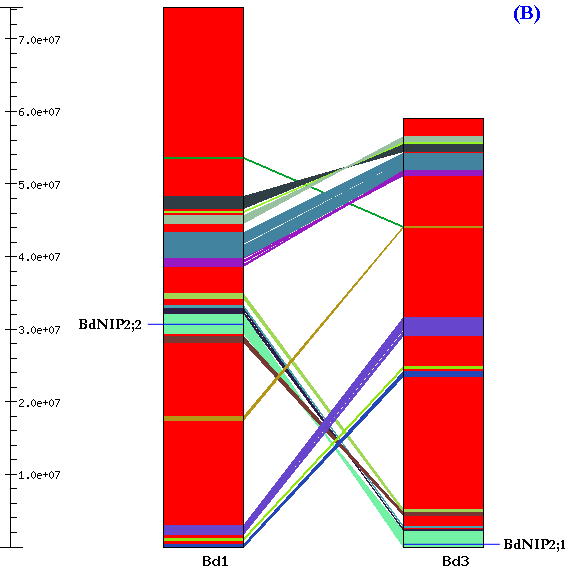


**Additional file 3**

Distribution of *NIP2* genes and segmental duplication events contributed to the evolution of *NIP* III subgroup in sorghum (A) and *Brachypodium* (B). The gene coordination files and predicted amino acid sequences for the sorghum and *Brachypodium* genomes were downloaded from Phytozome (http://www.phytozome.net). The predicted amino acid sequences were separated into different chromosomes according to the annotation information derived from the gene coordination files. The amino acid sequences within each chromosome were searched against each other using BLASTP. The hits with an E value less than 1e-5 were used as input for the Blast Synteny Toolkit (version 06132003) that was downloaded from TIGR, with the default parameters to generate the corresponding syntenic figures.
