# Supplementary material for: Functional divergence of the NIP III subgroup proteins involved altered selective constraints and positive selection
Source: BMC Plant Biol. 2010 Nov 20;10:256. doi: 10.1186/1471-2229-10-256 (PMC3095335; doi:10.1186/1471-2229-10-256)
Supplement: Additional file 4 — The exon/intron lengths and gene structure of NIP2 genes in monocot and dicot plants. [file 1471-2229-10-256-S4.DOC]

**Additional file 4**

The exon/intron lengths and gene structure of *NIP2* genes in monocot and dicot plants

| Species | NIP2s | Exon1 | Intron1 | Exon2 | Intron2 | Exon3 | Intron3 | Exon4 | Intron4 | Exon5 | Gene structure |
| --- | --- | --- | --- | --- | --- | --- | --- | --- | --- | --- | --- |
| O. sativa | OsNIP2;1 | 149 | 143 | 224 | 1606 | 194 | 105 | 61 | 350 | 264 | 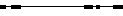 |
|  | OsNIP2;2 | 158 | 159 | 224 | 2094 | 194 | 113 | 61 | 713 | 255 | 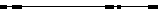 |
| *S. bicolor* | SbNIP2;1 | 146 | 158 | 224 | 1753 | 194 | 105 | 61 | 458 | 264 | 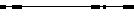 |
|  | SbNIP2;2 | 155 | 277 | 224 | 2257 | 194 | 129 | 61 | 755 | 249 | 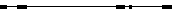 |
| *B. distachyon* | BdNIP2;1 | 149 | 121 | 224 | 1605 | 194 | 110 | 61 | 587 | 258 | 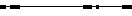 |
|  | BdNIP2;2 | 170 | 112 | 224 | 1872 | 194 | 117 | 61 | 674 | 255 | 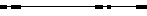 |
| *Z. mays* | ZmNIP2;1 | 143 | 156 | 224 | 1581 | 194 | 113 | 61 | 370 | 261 | 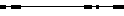 |
|  | ZmNIP2;2 | 158 | 134 | 224 | 2036 | 194 | 124 | 61 | 98 | 243 | 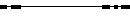 |
|  | ZmNIP2;3 | 167 | 120 | 224 | 1386 | 194 | 117 | 61 | 564 | 255 | 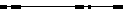 |
|  |  |  |  |  |  |  |  |  |  |  |  |
| *C. sativus* | CsNIP2;1 | 147 | 263 | 225 | 2336 | 195 | 348 | 62 | 342 | 238 | 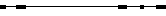 |
|  | CsNIP2;2 | 123 | 82 | 225 | 1741 | 195 | 97 | 62 | 192 | 181 | 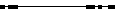 |
| *G. max* | GmNIP2;2 | 149 | 264 | 224 | 2926 | 194 | 222 | 61 | 224 | 249 | 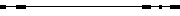 |
|  | GmNIP2;1 | 155 | 373 | 224 | 3068 | 194 | 208 | 61 | 225 | 249 | 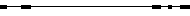 |
| *R. communis* | RcNIP2;1 | 155 | 158 | 224 | 911 | 194 | 103 | 61 | 536 | 255 | 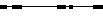 |
| *C. papaya* | CpaNIP2;1 | 140 | 89 | 224 | 1327 | 194 | 82 | 61 | 239 | 255 | 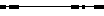 |
| *V. vinifera* | VvNIP2;1 | 146 | 260 | 224 | 1040 | 194 | 83 | 61 | 82 | 255 | 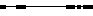 |
| *L. esculentum* | LeNIP2;1 | 118 | 79 | 228 | 5304 | 199 | 743 | 62 | 501 | 250 | 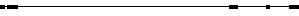 |
| *M. truncatula* | MtNIP2;1 | 116 | 94 | 230 | 461 | 199 | 357 | 62 | 291 | 208 | 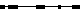 |
| *P. trichocarpa* | PtNIP2;1 | 102 | 253 | 225 | 1465 | 195 | 152 | 61 | 584 | 253 | 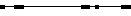 |
