# Supplementary material for: Functional divergence of the NIP III subgroup proteins involved altered selective constraints and positive selection
Source: BMC Plant Biol. 2010 Nov 20;10:256. doi: 10.1186/1471-2229-10-256 (PMC3095335; doi:10.1186/1471-2229-10-256)
Supplement: Additional file 5 — Spatial organization of residues forming the ar/R filter of the proximal part (located at the extracellular face) of the water channel. (A) Cucumber CsNIP2;1; (B) Cucumber CsNIP2;2; (C) Zucchini CpNIP2;1; (D) Barley HvNIP2;1; (E) Rice OsNIP2;1. The ar/R selectivity filter of NIP III proteins in dicot and monocot plants is composed of G, S, G, and R, with only one exception (CsNIP2;2), where the first residue in H2 was replaced by the bulkier Cys (C). [file 1471-2229-10-256-S5.DOC]

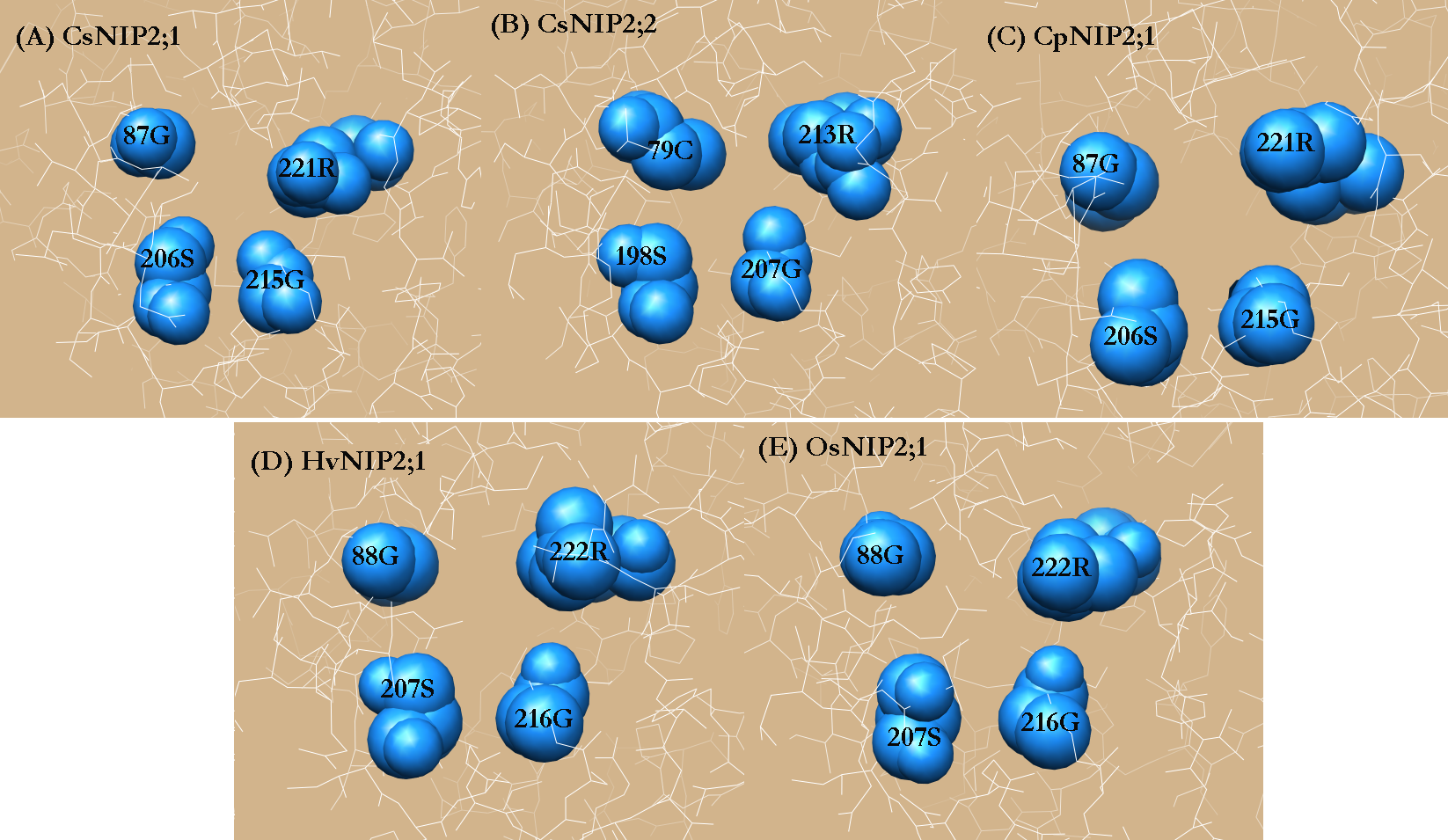


**Additional file 5**

Spatial organization of residues forming the ar/R filter of the proximal part (located at the extracellular face) of the water channel. (A) Cucumber CsNIP2;1; (B) Cucumber CsNIP2;2; (C) Zucchini CpNIP2;1; (D) Barley HvNIP2;1; (E) Rice OsNIP2;1. The ar/R selectivity filter of NIP III proteins in dicot and monocot plants is composed of G, S, G, and R, with only one exception (CsNIP2;2), where the first residue in H2 was replaced by the bulkier Cys (C).
