# Supplementary material for: Functional divergence of the NIP III subgroup proteins involved altered selective constraints and positive selection
Source: BMC Plant Biol. 2010 Nov 20;10:256. doi: 10.1186/1471-2229-10-256 (PMC3095335; doi:10.1186/1471-2229-10-256)
Supplement: Additional file 6 — Illustration of specificity determining positions (SDPs) in monocot and dicot plants. In the multiple-alignment of full-length NIP III protein sequences, the residues are displayed in the "Difference Mode" with the "Diff/Consensus Line" style. Dots indicate conserved residues with the first protein HvNIP2;1, and "-" indicates gaps on the alignment. The possible Specificity Determining Positions (SDPs) that might determine the functional specificity of orthologous NIP2 proteins after the monocot-dicot split are shaded in green. The dual NPA motifs are boxed. The four residues making up the ar/R filter are designated with arrows and highlighted in red. [file 1471-2229-10-256-S6.DOC]

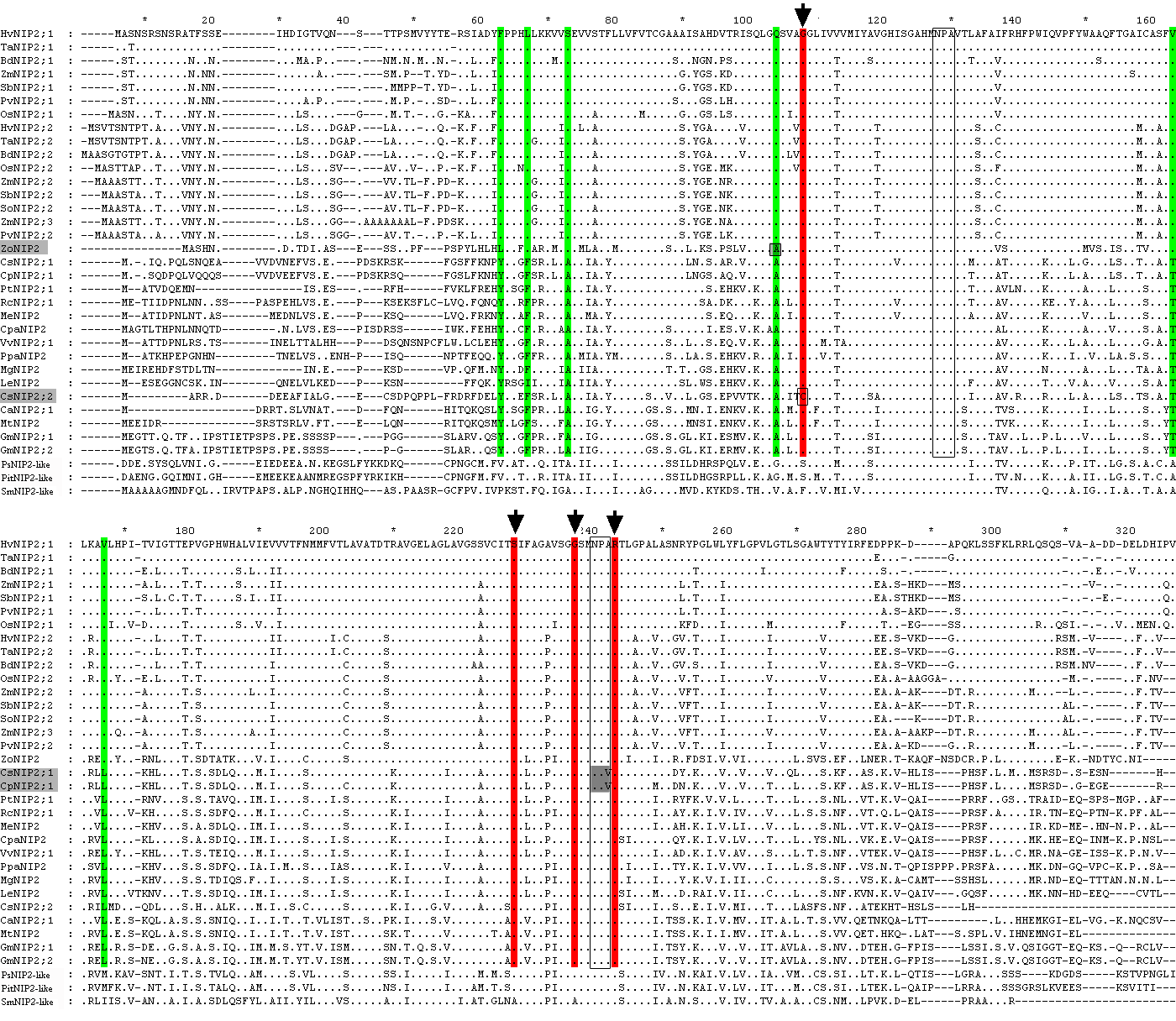


**Additional file 6**

Illustration of specificity determining positions (SDPs) in monocot and dicot plants. In the multiple-alignment of full-length NIP III protein sequences, the residues are displayed in the “Difference Mode” with the “Diff/Consensus Line” style. Dots indicate conserved residues with the first protein HvNIP2;1, and “−” indicates gaps on the alignment. The possible Specificity Determining Positions (SDPs) that might determine the functional specificity of orthologous NIP2 proteins after the monocot-dicot split are shaded in green. The dual NPA motifs are boxed. The four residues making up the ar/R filter are designated with arrows and highlighted in red.
