# Supplementary material for: Functional divergence of the NIP III subgroup proteins involved altered selective constraints and positive selection
Source: BMC Plant Biol. 2010 Nov 20;10:256. doi: 10.1186/1471-2229-10-256 (PMC3095335; doi:10.1186/1471-2229-10-256)

**Additional file 7**

The predicted serine and threonine phosphorylation sites in plant NIP III proteins

298 OsNIP2;1

MASNNSRTNSRANYSNEIHDLSTVQNGTMPTMYYGEKAIADFFPPHLLKKVVSEVVATFLLVFMTCGAAGISGSDLSRIS 80

QLGQSIAGGLIVTVMIYAVGHISGAHMNPAVTLAFAVFRHFPWIQVPFYWAAQFTGAICASFVLKAVIHPVDVIGTTTPV 160

GPHWHSLVVEVIVTFNMMFVTLAVATDTRAVGELAGLAVGSAVCITSIFAGAISGGSMNPARTLGPALASNKFDGLWIYF 240

LGPVMGTLSGAWTYTFIRFEDTPKEGSSQKLSSFKLRRLRSQQSIAADDVDEMENIQV 320

.....S...S....S........................................................S....S..S 80

...........................................................................T.T.. 160

..............................................................T................. 240

.....................T.....S...SS......................... 320

Serine predictions

Name Pos Context Score Pred

_________________________v_________________

OsNIP2;1 6 ASNNSRTNS 0.961 *S*

OsNIP2;1 10 SRTNSRANY 0.704 *S*

OsNIP2;1 15 RANYSNEIH 0.699 *S*

OsNIP2;1 72 AAGISGSDL 0.967 *S*

OsNIP2;1 77 GSDLSRISQ 0.889 *S*

OsNIP2;1 80 LSRISQLGQ 0.972 *S*

OsNIP2;1 268 KEGSSQKLS 0.984 *S*

OsNIP2;1 272 SQKLSSFKL 0.654 *S*

OsNIP2;1 273 QKLSSFKLR 0.942 *S*

_________________________^_________________

Threonine predictions

Name Pos Context Score Pred

_________________________v_________________

OsNIP2;1 156 DVIGTTTPV 0.647 *T*

OsNIP2;1 158 IGTTTPVGP 0.979 *T*

OsNIP2;1 223 NPARTLGPA 0.695 *T*

OsNIP2;1 262 RFEDTPKEG 0.997 *T*

_________________________^_________________


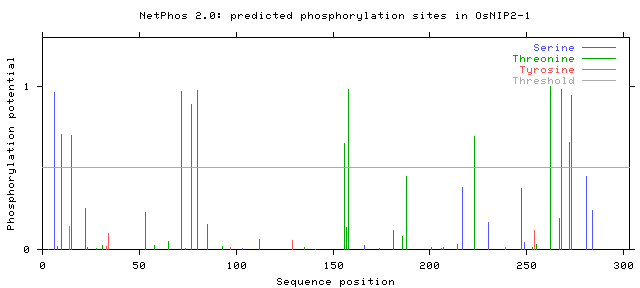


298 OsNIP2;2

MASTTAPSRTNSRVNYSNEIHDLSTVQSVSAVPSVYYPEKSFADIFPPNLLKKVISEVVATFLLVFVTCGAASIYGEDMK 80

RISQLGQSVVGGLIVTVMIYATGHISGAHMNPAVTLSFAFFRHFPWIQVPFYWAAQFTGAMCAAFVLRAVLYPIEVLGTT 160

TPTGPHWHALVIEIVVTFNMMFVTCAVATDSRAVGELAGLAVGSAVCITSIFAGPVSGGSMNPARTLAPAVASNVYTGLW 240

IYFLGPVVGTLSGAWVYTYIRFEEAPAAAGGAAPQKLSSFKLRRLQSQSMAADEFDNV 320

.......S...S....S......S.....S..........S...............................S....... 80

..S....S........................................................................ 160

T..........................................................S.....T.............. 240

.........T............................S.......S........... 320

Serine predictions

Name Pos Context Score Pred

_________________________v_________________

OsNIP2;2 8 TTAPSRTNS 0.988 *S*

OsNIP2;2 12 SRTNSRVNY 0.947 *S*

OsNIP2;2 17 RVNYSNEIH 0.820 *S*

OsNIP2;2 24 IHDLSTVQS 0.623 *S*

OsNIP2;2 30 VQSVSAVPS 0.759 *S*

OsNIP2;2 41 YPEKSFADI 0.935 *S*

OsNIP2;2 73 CGAASIYGE 0.971 *S*

OsNIP2;2 83 MKRISQLGQ 0.980 *S*

OsNIP2;2 88 QLGQSVVGG 0.555 *S*

OsNIP2;2 220 VSGGSMNPA 0.539 *S*

OsNIP2;2 279 QKLSSFKLR 0.864 *S*

OsNIP2;2 287 RRLQSQSMA 0.840 *S*

_________________________^_________________

Threonine predictions

Name Pos Context Score Pred

_________________________v_________________

OsNIP2;2 161 LGTTTPTGP 0.880 *T*

OsNIP2;2 226 NPARTLAPA 0.550 *T*

OsNIP2;2 250 PVVGTLSGA 0.513 *T*

_________________________^_________________


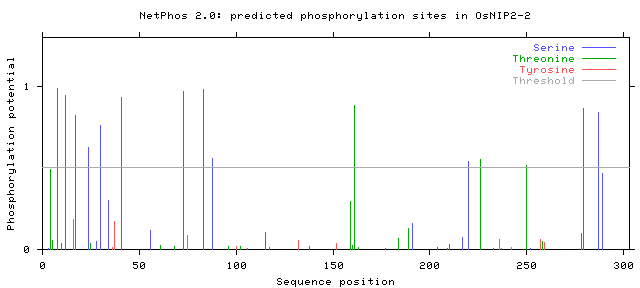


296 BdNIP2;1

MSTNSRSNSRANFSNEIHDMATPQNSNMPNMMYYNERSLADFFPPHLLKKMVSEVVSTFLLVFVTCGASAINGNDPSRIS 80

QLGQSVAGGLIVTVMIYSVGHISGAHMNPAVTTAFAVFRHFPWIQVPFYWASQFTGAICASFVLKAVLHPIEVLGTTTPV 160

GPHWHSLLIEIIVTFNMMFVTLAVATDTRAVGELAGLAVGSSVCITSIFAGAVSGGSMNPARTLGPALASNRYTGLWLYF 240

LGPILGTLSGAWTYTFIRFEDSPKDAPQKLSSFKLRRLQSQSVAAEDDDVLDHIPV 320

....S...S....S.......................S.........................................S 80

................................T............................................T.. 160

........................................................S.....T................. 240

.....................S.........S.......S.S.............. 320

Serine predictions

Name Pos Context Score Pred

_________________________v_________________

BdNIP2;1 5 MSTNSRSNS 0.871 *S*

BdNIP2;1 9 SRSNSRANF 0.871 *S*

BdNIP2;1 14 RANFSNEIH 0.855 *S*

BdNIP2;1 38 YNERSLADF 0.628 *S*

BdNIP2;1 80 PSRISQLGQ 0.975 *S*

BdNIP2;1 217 VSGGSMNPA 0.517 *S*

BdNIP2;1 262 RFEDSPKDA 0.998 *S*

BdNIP2;1 272 QKLSSFKLR 0.864 *S*

BdNIP2;1 280 RRLQSQSVA 0.582 *S*

BdNIP2;1 282 LQSQSVAAE 0.507 *S*

_________________________^_________________

Threonine predictions

Name Pos Context Score Pred

_________________________v_________________

BdNIP2;1 113 PAVTTAFAV 0.715 *T*

BdNIP2;1 158 LGTTTPVGP 0.956 *T*

BdNIP2;1 223 NPARTLGPA 0.695 *T*

_________________________^_________________


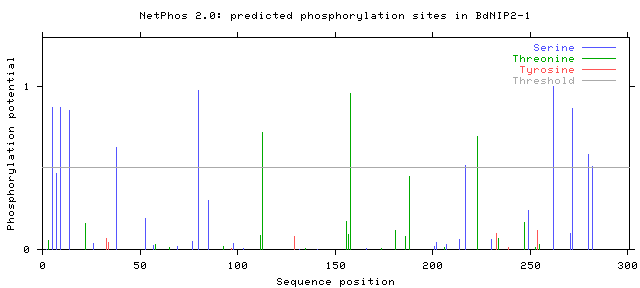


302 BdNIP2;2

MAASGTGTPTRANSRVNYSNEIHDLSTVQDGAPSLAPSMYYQEKSFADFFPPHLLKKVISEVVATFLLVFVTCGAASIYG 80

ADVTRVSQLGQSLVGGLIVTVMIYATGHISGAHMNPAVTLSFACFRHFPWIQVPFYWAAQFTGAMCAAFVLRAVLHPITV 160

LGTTTPTGPHWHALVIEIVVTFNMMFVTCAVATDSRAVGELAGLAVGAAVCITSIFAGPVSGGSMNPARTLAPAVASGVY 240

SGLWIYFLGPVIGTLSGAWVYTYIRFEEAPSVKDGPQKLSSFKLRRLQSQRSMANVDEFDHV 320

.......T.....S....S..................S......S...............................S... 80

......S......................................................................... 160

....T..........................................................S.....T.......... 240

.............T................S.........S.......S..S.......... 320

Serine predictions

Name Pos Context Score Pred

_________________________v_________________

BdNIP2;2 14 TRANSRVNY 0.978 *S*

BdNIP2;2 19 RVNYSNEIH 0.820 *S*

BdNIP2;2 38 SLAPSMYYQ 0.903 *S*

BdNIP2;2 45 YQEKSFADF 0.846 *S*

BdNIP2;2 77 CGAASIYGA 0.974 *S*

BdNIP2;2 87 VTRVSQLGQ 0.973 *S*

BdNIP2;2 224 VSGGSMNPA 0.539 *S*

BdNIP2;2 271 EEAPSVKDG 0.998 *S*

BdNIP2;2 281 QKLSSFKLR 0.864 *S*

BdNIP2;2 289 RRLQSQRSM 0.901 *S*

BdNIP2;2 292 QSQRSMANV 0.629 *S*

_________________________^_________________

Threonine predictions

Name Pos Context Score Pred

_________________________v_________________

BdNIP2;2 8 SGTGTPTRA 0.929 *T*

BdNIP2;2 165 LGTTTPTGP 0.880 *T*

BdNIP2;2 230 NPARTLAPA 0.550 *T*

BdNIP2;2 254 PVIGTLSGA 0.515 *T*

_________________________^_________________


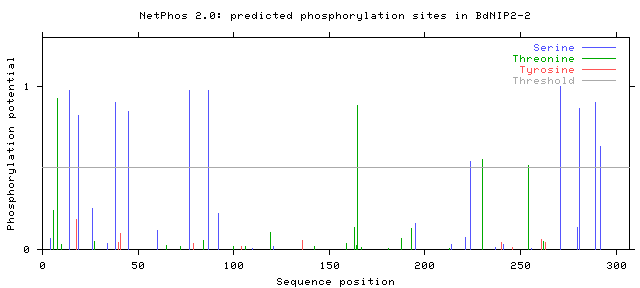


273 CaNIP2;1

MDRRTHSLVNATNDFQNHITQKQSLYPSGFPRKVLAEVIGTYLLVFVGSGSAAMNAIDENKVSKLGASMAGGFIVTVMIY 80

AIGHISGAHMNPAVSLAFATVSHFPWKQVPFYIAAQLTGAISASYTLKVLLEPSKQLGATSPSGSNIQALIIEIVTTFTM 160

VLISTAVSTDPKAIGELSGVAVGSSVCIASIVAGPISGGSMNPARTLGPAIATSSYKGIWVYMVGPITGALLGTWSYVVI 240

QETNKQALTTSLKLHHEMKGIELVGDKDNQCSV 320

......S................S...........................................S............ 80

.............................................T..............S...S............... 160

.......S.....................................T........S......................... 240

..T.............................. 320

Serine predictions

Name Pos Context Score Pred

_________________________v_________________

CaNIP2;1 7 RRTHSLVNA 0.986 *S*

CaNIP2;1 24 TQKQSLYPS 0.512 *S*

CaNIP2;1 68 KLGASMAGG 0.683 *S*

CaNIP2;1 141 LGATSPSGS 0.807 *S*

CaNIP2;1 145 SPSGSNIQA 0.598 *S*

CaNIP2;1 168 STAVSTDPK 0.762 *S*

CaNIP2;1 215 IATSSYKGI 0.599 *S*

_________________________^_________________

Threonine predictions

Name Pos Context Score Pred

_________________________v_________________

CaNIP2;1 126 SASYTLKVL 0.633 *T*

CaNIP2;1 206 NPARTLGPA 0.695 *T*

CaNIP2;1 243 VIQETNKQA 0.505 *T*

_________________________^_________________


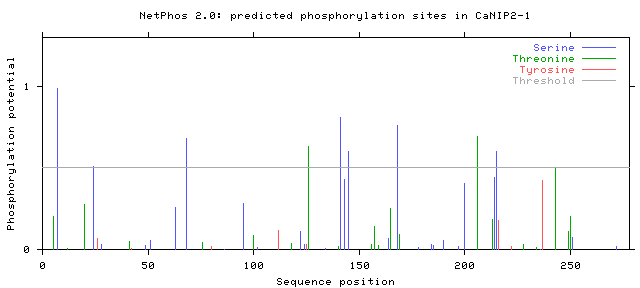


288 CpNIP2;1

MSSSQDPQLVQQQSVVDVEEFVSVENPDSKRSQFGSLFKNHYPPGFSRKLVAEVIATYLLVFVTCGAAALNGSDAQRVSQ 80

LGASVAGGLIVTVMIYAVGHISGAHMNPAVTTAFAATRHFPWKQVPLYAAAQLSGATCAAFTLRLLLHPIKHLGTTTPSG 160

SDLQALVMEIVVTFSMMFVTCAVATDTKAVGELAGLAVGSAVCITSILAGPVSGGSMNPVRTLGPAMASDNYKGLWVYFV 240

GPVTGTLLGAWSYKFIRASDKPVHLISPHSFSLKLRRMSRSDVGEGER 320

.............S........S.....S..S..............................................S. 80

...............................T.............................T..............T.S. 160

..........................T..................................T.................. 240

...........S......S.......S....S......S.S....... 320

Serine predictions

Name Pos Context Score Pred

_________________________v_________________

CpNIP2;1 14 VQQQSVVDV 0.763 *S*

CpNIP2;1 23 EEFVSVENP 0.971 *S*

CpNIP2;1 29 ENPDSKRSQ 0.974 *S*

CpNIP2;1 32 DSKRSQFGS 0.907 *S*

CpNIP2;1 79 AQRVSQLGA 0.885 *S*

CpNIP2;1 159 TTTPSGSDL 0.978 *S*

CpNIP2;1 252 LGAWSYKFI 0.694 *S*

CpNIP2;1 259 FIRASDKPV 0.964 *S*

CpNIP2;1 267 VHLISPHSF 0.573 *S*

CpNIP2;1 272 PHSFSLKLR 0.744 *S*

CpNIP2;1 279 LRRMSRSDV 0.998 *S*

CpNIP2;1 281 RMSRSDVGE 0.641 *S*

_________________________^_________________

Threonine predictions

Name Pos Context Score Pred

_________________________v_________________

CpNIP2;1 112 PAVTTAFAA 0.726 *T*

CpNIP2;1 142 CAAFTLRLL 0.650 *T*

CpNIP2;1 157 LGTTTPSGS 0.912 *T*

CpNIP2;1 187 VATDTKAVG 0.670 *T*

CpNIP2;1 222 NPVRTLGPA 0.655 *T*

_________________________^_________________


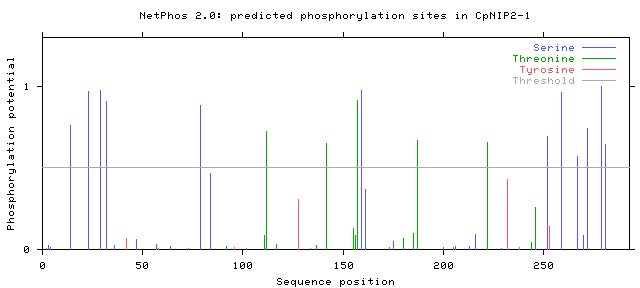


292 CpaNIP2

MAGTLTHPNLNNQTDINDLVSVESPISDRSSIWKSFEHHYPPCFLRKVAAEVIATYLLVFVTCGSAAISAIDESRVSKLA 80

ASVAGGLIVTVMIYAVGHISGAHMNPAVTLAFAALRHFPWKQVPFYAAAQVTGAISAAFTLRVLLHPIKLIGTTSPAGSD 160

IQALIMEIVVTFSMMFITSAVATDTKAVGELAGIAVGSAVCITSILAGPVSGGSMNPARSIGPALASQYYKGIWVYLVGP 240

VTGTLLGAYSYNLIRVKDEPVQAISPRSFSFKLRRMKSHEEQINMKDPLNSL 320

....................S..S..S..SS...S.................................S.......S... 80

.S.........................................................T..............S...S. 160

........................T............................S.....S.................... 240

........................S....S.......S.............. 320

Serine predictions

Name Pos Context Score Pred

_________________________v_________________

CpaNIP2 21 NDLVSVESP 0.989 *S*

CpaNIP2 24 VSVESPISD 0.975 *S*

CpaNIP2 27 ESPISDRSS 0.968 *S*

CpaNIP2 30 ISDRSSIWK 0.690 *S*

CpaNIP2 31 SDRSSIWKS 0.990 *S*

CpaNIP2 35 SIWKSFEHH 0.738 *S*

CpaNIP2 69 SAAISAIDE 0.991 *S*

CpaNIP2 77 ESRVSKLAA 0.989 *S*

CpaNIP2 82 KLAASVAGG 0.756 *S*

CpaNIP2 155 IGTTSPAGS 0.731 *S*

CpaNIP2 159 SPAGSDIQA 0.923 *S*

CpaNIP2 214 VSGGSMNPA 0.539 *S*

CpaNIP2 220 NPARSIGPA 0.914 *S*

CpaNIP2 265 VQAISPRSF 0.973 *S*

CpaNIP2 270 PRSFSFKLR 0.982 *S*

CpaNIP2 278 RRMKSHEEQ 0.998 *S*

_________________________^_________________

Threonine predictions

Name Pos Context Score Pred

_________________________v_________________

CpaNIP2 140 SAAFTLRVL 0.929 *T*

CpaNIP2 185 VATDTKAVG 0.670 *T*

_________________________^_________________


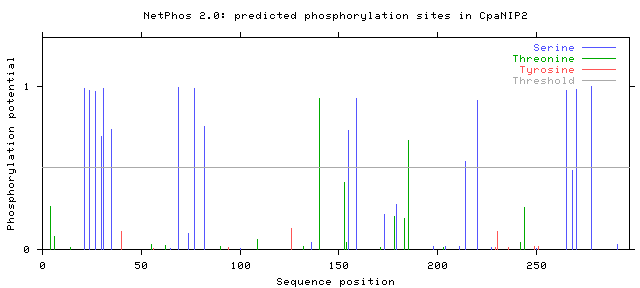


288 CsNIP2;1

MSSIQNPQLSNQEAVVDVNEFVSVENPDSKRSKFGSFFKNPYPPGFSRKLVAEVIATYLLVFVTCGAAALNASDARRVSQ 80

LGASVAGGLIVTVMIYAVGHVSGAHMNPAVTMAFAATRHFPWKQVPLYGAAQLSGATCAAFTLRLLLHPIKHLGTTTPSG 160

SDLQALVMEIVVTFSMMFVTLAVATDTKAVGELAGIAVGSAVCITSILAGPVSGGSMNPVRTLGPALASDYYKGLWVYFV 240

GPVVGTQLGAWSYKFIRASDKPVHLISPHSFSLKMRRMSRSDVSESNH 320

.........S............S.....S..S..............................................S. 80

.............................................................T..............T.S. 160

..........................T..................................T.........Y........ 240

...........S......S.......S....S......S.S..S.... 320

Serine predictions

Name Pos Context Score Pred

_________________________v_________________

CsNIP2;1 10 NPQLSNQEA 0.704 *S*

CsNIP2;1 23 NEFVSVENP 0.981 *S*

CsNIP2;1 29 ENPDSKRSK 0.890 *S*

CsNIP2;1 32 DSKRSKFGS 0.951 *S*

CsNIP2;1 79 ARRVSQLGA 0.994 *S*

CsNIP2;1 159 TTTPSGSDL 0.978 *S*

CsNIP2;1 252 LGAWSYKFI 0.763 *S*

CsNIP2;1 259 FIRASDKPV 0.964 *S*

CsNIP2;1 267 VHLISPHSF 0.573 *S*

CsNIP2;1 272 PHSFSLKMR 0.962 *S*

CsNIP2;1 279 MRRMSRSDV 0.998 *S*

CsNIP2;1 281 RMSRSDVSE 0.770 *S*

CsNIP2;1 284 RSDVSESNH 0.989 *S*

_________________________^_________________

Threonine predictions

Name Pos Context Score Pred

_________________________v_________________

CsNIP2;1 142 CAAFTLRLL 0.650 *T*

CsNIP2;1 157 LGTTTPSGS 0.912 *T*

CsNIP2;1 187 VATDTKAVG 0.670 *T*

CsNIP2;1 222 NPVRTLGPA 0.655 *T*

_________________________^_________________


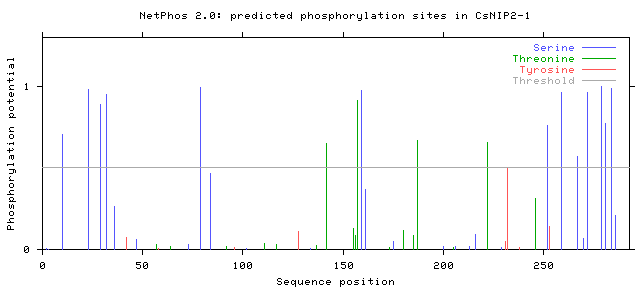


261 CsNIP2;2

MARRSDDEEAFIALGNECSDPQPPLFRDRFDELYPPEFSRKLVAEVIATYLLVFVSCGVAALSGSDEPVVTKLGASITCG 80

LIVTVMIYSAGHISGAHMNPAVTIAFAAVRRFPWRQVPLYAAAQLSGATSAAFTLRILMDPIQDLGTTSPHGPALKALVM 160

EIVVSFCMMFVTSAVATDTKAIGELGGVAVGSAVCISSIFAGPISGGSMNPARSIGPAIASSRYEGIWVYMIGPVTGTLL 240

ASFSYNFIRATEKHTHSLSLH 320

....S.........................................................S............S.... 80

.....................................................T..............S........... 160

............S........................................S.......S.................. 240

..........T.......... 320

Serine predictions

Name Pos Context Score Pred

_________________________v_________________

CsNIP2;2 5 MARRSDDEE 0.993 *S*

CsNIP2;2 63 VAALSGSDE 0.995 *S*

CsNIP2;2 76 KLGASITCG 0.897 *S*

CsNIP2;2 149 LGTTSPHGP 0.915 *S*

CsNIP2;2 173 MFVTSAVAT 0.655 *S*

CsNIP2;2 214 NPARSIGPA 0.914 *S*

CsNIP2;2 222 AIASSRYEG 0.977 *S*

_________________________^_________________

Threonine predictions

Name Pos Context Score Pred

_________________________v_________________

CsNIP2;2 134 SAAFTLRIL 0.747 *T*

CsNIP2;2 251 FIRATEKHT 0.949 *T*

_________________________^_________________


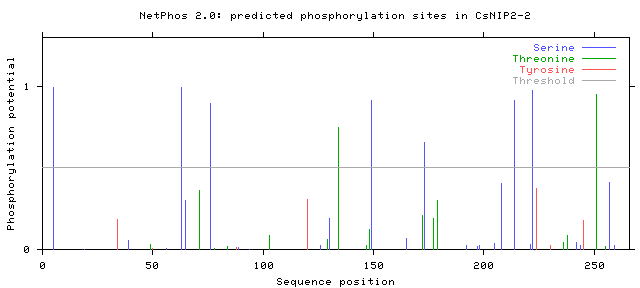


295 GmNIP2;1

MEGTTSQSTFTFIPSTIETPSPSIPEISSSSPSPGGSLARVAQSYPPGFPRKVFAEVIGTFLLVFVGSGSAGLSKIDESM 80

VSKLGASLAGGLIVTVMIYSIGHISGAHMNPAVSLAFTAVRHLPWPQLPFYVAAQLTGAISASYTLRELLRPSDEIGGTS 160

PAGSHIQALIMEMVSTYTMVFISMAVATDSNATGQLSGVAVGSSVCIASIVAGPISGGSMNPARTLGPAIATSYYKGLWV 240

YFVGPITGAVLAAWSYNVIRDTEHPGFPISLSSISSKVRQSIGGTEQKSDQRCLV 320

...T..........S...T.S.S.....S.S.S..........S.............................S....S. 80

.................................S..............................T.......S......S 160

...S............................................................T.......S....... 240

.....................T.........SS.SS....S.............. 320

Serine predictions

Name Pos Context Score Pred

_________________________v_________________

GmNIP2;1 15 TFIPSTIET 0.974 *S*

GmNIP2;1 21 IETPSPSIP 0.975 *S*

GmNIP2;1 23 TPSPSIPEI 0.913 *S*

GmNIP2;1 29 PEISSSSPS 0.660 *S*

GmNIP2;1 31 ISSSSPSPG 0.988 *S*

GmNIP2;1 33 SSSPSPGGS 0.995 *S*

GmNIP2;1 44 RVAQSYPPG 0.512 *S*

GmNIP2;1 74 SAGLSKIDE 0.993 *S*

GmNIP2;1 79 KIDESMVSK 0.593 *S*

GmNIP2;1 114 NPAVSLAFT 0.595 *S*

GmNIP2;1 153 LLRPSDEIG 0.975 *S*

GmNIP2;1 160 IGGTSPAGS 0.770 *S*

GmNIP2;1 164 SPAGSHIQA 0.952 *S*

GmNIP2;1 233 AIATSYYKG 0.697 *S*

GmNIP2;1 272 PISLSSISS 0.504 *S*

GmNIP2;1 273 ISLSSISSK 0.501 *S*

GmNIP2;1 275 LSSISSKVR 0.679 *S*

GmNIP2;1 276 SSISSKVRQ 0.675 *S*

GmNIP2;1 281 KVRQSIGGT 0.996 *S*

_________________________^_________________

Threonine predictions

Name Pos Context Score Pred

_________________________v_________________

GmNIP2;1 4 -MEGTTSQS 0.635 *T*

GmNIP2;1 19 STIETPSPS 0.929 *T*

GmNIP2;1 145 SASYTLREL 0.934 *T*

GmNIP2;1 225 NPARTLGPA 0.695 *T*

GmNIP2;1 262 VIRDTEHPG 0.892 *T*

_________________________^_________________


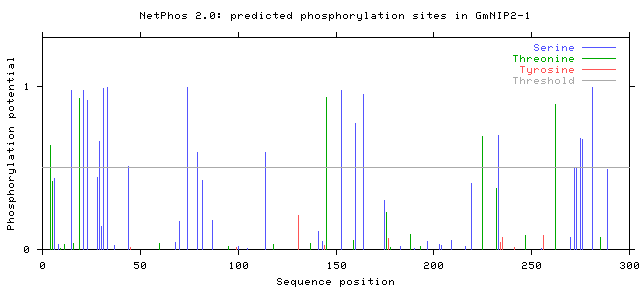


293 GmNIP2;2

MEGTSSQSTFAFIPSTIETPSPSIPEISSSSSPGSLARIAQSYPPGFPRKVLAEIIGTFLLVFVGSGSAGLSKIDERMVS 80

KLGASLAGGLIVTVMIYSIGHISGAHMNPAVSLAFTAVRHLPWPQLPFYIAAQLTGAISASYTLRELLRPSNEIGGTSPA 160

GSHIQALIMEMVTTYTMVFISMAVATDSNATGQLSGVAVGSSVCIASIVAGPISGGSMNPARTLGPAIATSYYKGLWVYF 240

VGPITGAVLAAWSYNVIRDTEHPGFPISLSSISSKVRQSIGGTEQKSDQRCLV 320

...T..........S...T.S.S....S.SSS..S....................................S.......S 80

...............................S..............................T.......S......S.. 160

.S............................................................T.......S......... 240

...................T.........SS.SS....S.............. 320

Serine predictions

Name Pos Context Score Pred

_________________________v_________________

GmNIP2;2 15 AFIPSTIET 0.923 *S*

GmNIP2;2 21 IETPSPSIP 0.975 *S*

GmNIP2;2 23 TPSPSIPEI 0.913 *S*

GmNIP2;2 28 IPEISSSSS 0.759 *S*

GmNIP2;2 30 EISSSSSPG 0.745 *S*

GmNIP2;2 31 ISSSSSPGS 0.644 *S*

GmNIP2;2 32 SSSSSPGSL 0.995 *S*

GmNIP2;2 35 SSPGSLARI 0.858 *S*

GmNIP2;2 72 SAGLSKIDE 0.996 *S*

GmNIP2;2 80 ERMVSKLGA 0.901 *S*

GmNIP2;2 112 NPAVSLAFT 0.595 *S*

GmNIP2;2 151 LLRPSNEIG 0.889 *S*

GmNIP2;2 158 IGGTSPAGS 0.770 *S*

GmNIP2;2 162 SPAGSHIQA 0.952 *S*

GmNIP2;2 231 AIATSYYKG 0.697 *S*

GmNIP2;2 270 PISLSSISS 0.504 *S*

GmNIP2;2 271 ISLSSISSK 0.501 *S*

GmNIP2;2 273 LSSISSKVR 0.679 *S*

GmNIP2;2 274 SSISSKVRQ 0.675 *S*

GmNIP2;2 279 KVRQSIGGT 0.996 *S*

_________________________^_________________

Threonine predictions

Name Pos Context Score Pred

_________________________v_________________

GmNIP2;2 4 -MEGTSSQS 0.731 *T*

GmNIP2;2 19 STIETPSPS 0.929 *T*

GmNIP2;2 143 SASYTLREL 0.934 *T*

GmNIP2;2 223 NPARTLGPA 0.695 *T*

GmNIP2;2 260 VIRDTEHPG 0.892 *T*

_________________________^_________________


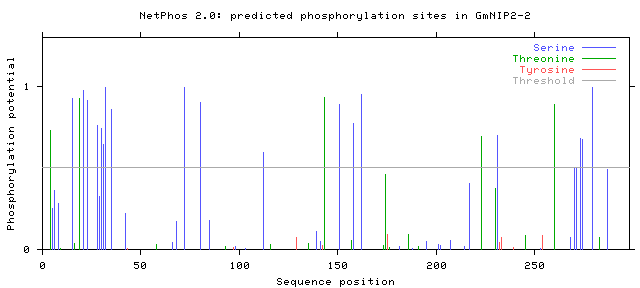


295 HvNIP2;1

MASNSRSNSRATFSSEIHDIGTVQNSTTPSMVYYTERSIADYFPPHLLKKVVSEVVSTFLLVFVTCGAAAISAHDVTRIS 80

QLGQSVAGGLIVVVMIYAVGHISGAHMNPAVTLAFAIFRHFPWIQVPFYWAAQFTGAICASFVLKAVLHPITVIGTTEPV 160

GPHWHALVIEVVVTFNMMFVTLAVATDTRAVGELAGLAVGSSVCITSIFAGAVSGGSMNPARTLGPALASNRYPGLWLYF 240

LGPVLGTLSGAWTYTYIRFEDPPKDAPQKLSSFKLRRLQSQSVAADDDELDHIPV 320

....S.S.S..T.S...........S.......Y...S.................................S.......S 80

................................................................................ 160

........................................................S.....T................. 240

...............................S.......S.S............. 320

Serine predictions

Name Pos Context Score Pred

_________________________v_________________

HvNIP2;1 5 MASNSRSNS 0.806 *S*

HvNIP2;1 7 SNSRSNSRA 0.834 *S*

HvNIP2;1 9 SRSNSRATF 0.510 *S*

HvNIP2;1 14 RATFSSEIH 0.932 *S*

HvNIP2;1 26 TVQNSTTPS 0.652 *S*

HvNIP2;1 38 YTERSIADY 0.912 *S*

HvNIP2;1 72 AAAISAHDV 0.780 *S*

HvNIP2;1 80 VTRISQLGQ 0.953 *S*

HvNIP2;1 217 VSGGSMNPA 0.517 *S*

HvNIP2;1 272 QKLSSFKLR 0.864 *S*

HvNIP2;1 280 RRLQSQSVA 0.582 *S*

HvNIP2;1 282 LQSQSVAAD 0.514 *S*

_________________________^_________________

Threonine predictions

Name Pos Context Score Pred

_________________________v_________________

HvNIP2;1 12 NSRATFSSE 0.571 *T*

HvNIP2;1 223 NPARTLGPA 0.695 *T*

_________________________^_________________


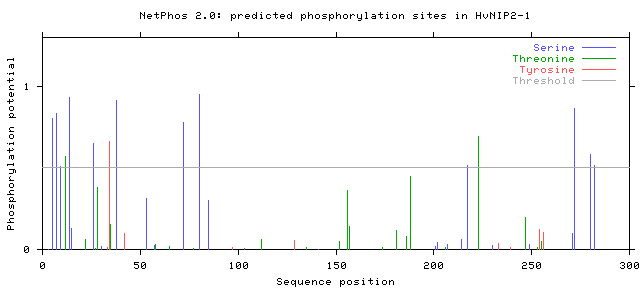


300 HvNIP2;2

MSVTSNTPTRANSRVNYSNEIHDLSTVQDGAPSLAPSMYYQEKSFADFFPPHLLKKVISELVATFLLVFVTCGAASIYGA 80

DVTRVSQLGQSVVGGLIVTVMIYATGHISGAHMNPAVTLSFACFRHFPWIQVPFYWAAQFTGAMCAAFVLRAVLHPITVL 160

GTTTPTGPHWHALVIEIIVTFNMMFITCAVATDSRAVGELAGLAVGSAVCITSIFAGPVSGGSMNPARTLAPAVASGVYT 240

GLWIYFLGPVIGTLSGAWVYTYIRFEEEPSVKDGPQKLSSFKLRRLQSQRSMAVDEFDHV 320

....S.T.....S....S..................S......S...............................S.... 80

.....S....S..................................................................... 160

...T..........................................................S.....T........... 240

............T................S.........S.......S..S......... 320

Serine predictions

Name Pos Context Score Pred

_________________________v_________________

HvNIP2;2 5 MSVTSNTPT 0.509 *S*

HvNIP2;2 13 TRANSRVNY 0.978 *S*

HvNIP2;2 18 RVNYSNEIH 0.820 *S*

HvNIP2;2 37 SLAPSMYYQ 0.903 *S*

HvNIP2;2 44 YQEKSFADF 0.846 *S*

HvNIP2;2 76 CGAASIYGA 0.974 *S*

HvNIP2;2 86 VTRVSQLGQ 0.973 *S*

HvNIP2;2 91 QLGQSVVGG 0.555 *S*

HvNIP2;2 223 VSGGSMNPA 0.539 *S*

HvNIP2;2 270 EEEPSVKDG 0.998 *S*

HvNIP2;2 280 QKLSSFKLR 0.864 *S*

HvNIP2;2 288 RRLQSQRSM 0.901 *S*

HvNIP2;2 291 QSQRSMAVD 0.631 *S*

_________________________^_________________

Threonine predictions

Name Pos Context Score Pred

_________________________v_________________

HvNIP2;2 7 VTSNTPTRA 0.790 *T*

HvNIP2;2 164 LGTTTPTGP 0.880 *T*

HvNIP2;2 229 NPARTLAPA 0.550 *T*

HvNIP2;2 253 PVIGTLSGA 0.515 *T*

_________________________^_________________


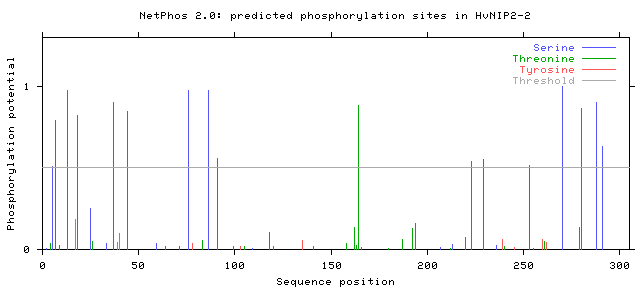


283 LeNIP2

MESEGGNCSKSINQNELVLKEDPKSNFFQKYYRSGIIKKVIAEIIATYLLVFVTCGAASLSWSDEHKVSKLGASVAGGLI 80

VTVMIYAVGHISGAHMNPAVTFAFAAVRHFPWTQVPVYAAAQVTGAISAAFTLRVLLHPVTKNVGTTTPSGSDIQALIME 160

IVVTFSMMFITSAVATDTKAIGELAGIAVGSAVCITSILAGPVSGGSMNPARSIGPAMASNDYRAIWVYIIGPVCGTLLG 240

AWSYNFIKVNDKPVQAIVPGQSFSFKLRRMKSNNHDEEQCVTL 320

..........S.............S.....Y...........................S.S.S.....S........... 80

................................T..................T...............T.S.S........ 160

..............................................S.....S........................... 240

........................................... 320

Serine predictions

Name Pos Context Score Pred

_________________________v_________________

LeNIP2 11 NCSKSINQN 0.735 *S*

LeNIP2 25 EDPKSNFFQ 0.893 *S*

LeNIP2 59 CGAASLSWS 0.970 *S*

LeNIP2 61 AASLSWSDE 0.994 *S*

LeNIP2 63 SLSWSDEHK 0.739 *S*

LeNIP2 69 EHKVSKLGA 0.634 *S*

LeNIP2 150 TTTPSGSDI 0.977 *S*

LeNIP2 152 TPSGSDIQA 0.841 *S*

LeNIP2 207 VSGGSMNPA 0.539 *S*

LeNIP2 213 NPARSIGPA 0.876 *S*

_________________________^_________________

Threonine predictions

Name Pos Context Score Pred

_________________________v_________________

LeNIP2 113 HFPWTQVPV 0.667 *T*

LeNIP2 132 SAAFTLRVL 0.929 *T*

LeNIP2 148 VGTTTPSGS 0.958 *T*

_________________________^_________________


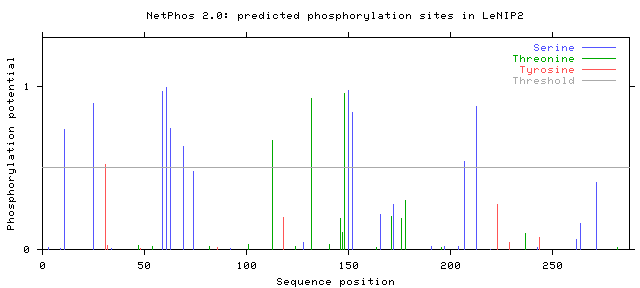


274 MtNIP2

MEEIDRSRSTSRLVTFTNELQNRITQKQSMYPLGFSKKVFAEVIGTYLLVFVGSGAAAMNSIDENKVSKLGASLAGGFIV 80

TVMIYAIGHISGAHMNPAVSLAFATVKHFPWKQVPFYIAAQLTGAISASYTLRVLLEPSKQLGATSPSGSNIQALIIEIV 160

TTFTMVFISTAVATDSKATGELAGVAVGSSVTIASIVAGPISGGSMNPARTLGPAIATSSYKGIWIYMVGPITGALLGAW 240

SYVVIQETDHKQDLATSQSPLSVKIHNEMNGIEL 320

......S.STS.............T...S...............................S................... 80

........................T.........................T..............S...S.......... 160

.........T........................................T........S.................... 240

................S....S............ 320

Serine predictions

Name Pos Context Score Pred

_________________________v_________________

MtNIP2 7 EIDRSRSTS 0.876 *S*

MtNIP2 9 DRSRSTSRL 0.997 *S*

MtNIP2 11 SRSTSRLVT 0.993 *S*

MtNIP2 29 TQKQSMYPL 0.582 *S*

MtNIP2 61 AAMNSIDEN 0.753 *S*

MtNIP2 146 LGATSPSGS 0.807 *S*

MtNIP2 150 SPSGSNIQA 0.598 *S*

MtNIP2 220 IATSSYKGI 0.599 *S*

MtNIP2 257 DLATSQSPL 0.508 *S*

MtNIP2 262 QSPLSVKIH 0.987 *S*

_________________________^_________________

Threonine predictions

Name Pos Context Score Pred

_________________________v_________________

MtNIP2 10 RSRSTSRLV 0.991 *T*

MtNIP2 25 QNRITQKQS 0.717 *T*

MtNIP2 105 LAFATVKHF 0.593 *T*

MtNIP2 131 SASYTLRVL 0.748 *T*

MtNIP2 170 VFISTAVAT 0.504 *T*

MtNIP2 211 NPARTLGPA 0.695 *T*

_________________________^_________________


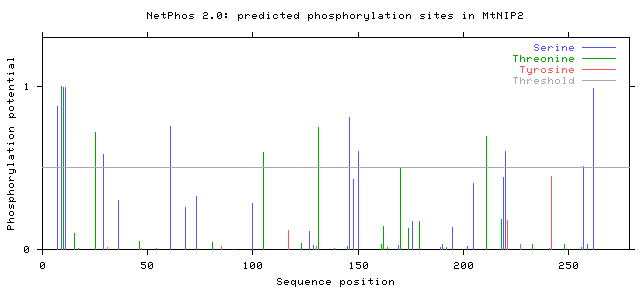


278 PtNIP2;1

MATVDQEMNISVESSRFHFVKLFREHYPSGFLRKVVAEVIATYLLVFVTCGAAAISASDEHKVSKLGASVAGGLIVTVMI 80

YAVGHISGAHMNPAVTTAFAAVLNFPWKQVPFYAAAQLTGAISASFTLKVLLHPIRNVGTTSPSGTAVQALIMEIVVTFS 160

MMFITSAVATDTKAVGELAGIAVGSAVCITSILAGPVSGGSMNPARTLGPAIASRYFKGVWVYLLGPVTGTLLGAWSYNL 240

IRVTDKPVQAIPRRFSFGSRRTRAIDEQSPSMGPLDAF 320

..........S..S.........................................S.S.....S................ 80

................T.............................T..............S.................. 160

...........T............................S.....T................................. 240

...T...........S............S.S....... 320

Serine predictions

Name Pos Context Score Pred

_________________________v_________________

PtNIP2;1 11 EMNISVESS 0.884 *S*

PtNIP2;1 14 ISVESSRFH 0.834 *S*

PtNIP2;1 56 AAAISASDE 0.976 *S*

PtNIP2;1 58 AISASDEHK 0.578 *S*

PtNIP2;1 64 EHKVSKLGA 0.634 *S*

PtNIP2;1 142 VGTTSPSGT 0.970 *S*

PtNIP2;1 201 VSGGSMNPA 0.539 *S*

PtNIP2;1 256 PRRFSFGSR 0.995 *S*

PtNIP2;1 269 IDEQSPSMG 0.966 *S*

PtNIP2;1 271 EQSPSMGPL 0.898 *S*

_________________________^_________________

Threonine predictions

Name Pos Context Score Pred

_________________________v_________________

PtNIP2;1 97 PAVTTAFAA 0.726 *T*

PtNIP2;1 127 SASFTLKVL 0.662 *T*

PtNIP2;1 172 VATDTKAVG 0.670 *T*

PtNIP2;1 207 NPARTLGPA 0.695 *T*

PtNIP2;1 244 LIRVTDKPV 0.748 *T*

_________________________^_________________


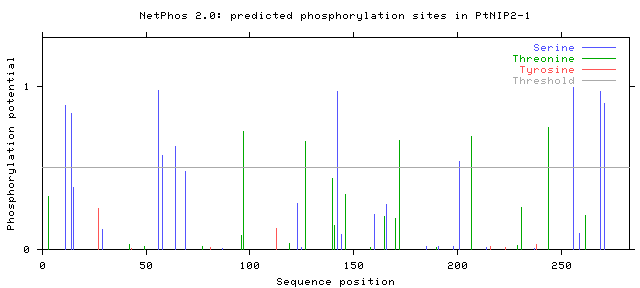


297 RcNIP2;1

METIIDPNLNNSSSSPASPEHLVSVENPKSEKSFLCLVQSFQNQYPPRFPRKVVAEVIATYLLVFVTCGAAAISSADDKR 80

ISKLGASLAGGLIVTVMIYAVGHVSGAHMNPAVTTAFAAVRHFPWKEVPYYAAAQLTGAISASFTLKVLLHPVKHIGTTS 160

PSGSDFQALVMEIVVTFCMMFVTSAVATDTKAIGELAGIAVGSAVCITSILAGPISGGSMNPARTLGPAIASAYYKGIWV 240

YIVGPVVGTLLGSWSYNFIRVTDQPLQAISPRSFSAKLRRIRSTNEQPTNKDPFDAL 320

...........S..S..S.....S.....S..S........................................SS..... 80

.S................................T..............YY.............T..............S 160

.S.S...................S........................................T............... 240

.............................S..S.S.......S.....T........ 320

Serine predictions

Name Pos Context Score Pred

_________________________v_________________

RcNIP2;1 12 NLNNSSSSP 0.547 *S*

RcNIP2;1 15 NSSSSPASP 0.986 *S*

RcNIP2;1 18 SSPASPEHL 0.997 *S*

RcNIP2;1 24 EHLVSVENP 0.988 *S*

RcNIP2;1 30 ENPKSEKSF 0.968 *S*

RcNIP2;1 33 KSEKSFLCL 0.506 *S*

RcNIP2;1 74 AAAISSADD 0.959 *S*

RcNIP2;1 75 AAISSADDK 0.807 *S*

RcNIP2;1 82 DKRISKLGA 0.985 *S*

RcNIP2;1 160 IGTTSPSGS 0.951 *S*

RcNIP2;1 162 TTSPSGSDF 0.989 *S*

RcNIP2;1 164 SPSGSDFQA 0.666 *S*

RcNIP2;1 184 MFVTSAVAT 0.655 *S*

RcNIP2;1 270 LQAISPRSF 0.934 *S*

RcNIP2;1 273 ISPRSFSAK 0.618 *S*

RcNIP2;1 275 PRSFSAKLR 0.985 *S*

RcNIP2;1 283 RRIRSTNEQ 0.987 *S*

_________________________^_________________

Threonine predictions

Name Pos Context Score Pred

_________________________v_________________

RcNIP2;1 115 PAVTTAFAA 0.726 *T*

RcNIP2;1 145 SASFTLKVL 0.662 *T*

RcNIP2;1 225 NPARTLGPA 0.695 *T*

RcNIP2;1 289 NEQPTNKDP 0.729 *T*


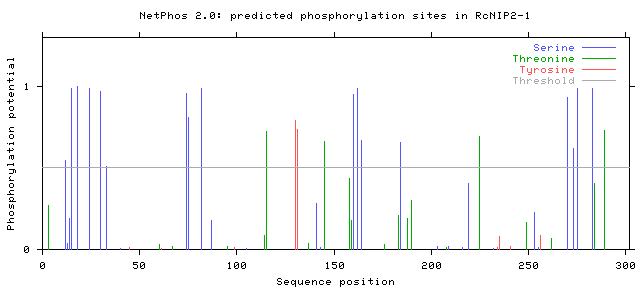


297 SbNIP2;1

MSTNSRSNSRANFNNEIHDIGTVQNSTMMPPTYYDRSLADIFPPHLLKKVVSEVVSTFLLVFVTCGAAGIYGSDKDRISQ 80

LGQSVAGGLIVTVMIYAVGHISGAHMNPAVTLAFAVFRHFPWIQVPFYWAAQFTGAICASFVLKAVLHPISVLGCTTPTG 160

PHWHSLIIEIIVTFNMMFVTLAVATDTRAVGELAGLAVGSAVCITSIFAGAVSGGSMNPARTLGPALASNLYTGLWIYFL 240

GPVLGTLSGAWTYTYIRFEEAPSTHKDMSQKLSSFKLRRLQSQSVAAEDDELDHIQV 320

....S....................S..........S...................................S.....S. 80

............................................................................T... 160

.......................................................S.....T.................. 240

......................ST....S...SS.......S.S............. 320

Serine predictions

Name Pos Context Score Pred

_________________________v_________________

SbNIP2;1 5 MSTNSRSNS 0.871 *S*

SbNIP2;1 26 TVQNSTMMP 0.775 *S*

SbNIP2;1 37 YYDRSLADI 0.926 *S*

SbNIP2;1 73 GIYGSDKDR 0.974 *S*

SbNIP2;1 79 KDRISQLGQ 0.987 *S*

SbNIP2;1 216 VSGGSMNPA 0.517 *S*

SbNIP2;1 263 EEAPSTHKD 0.855 *S*

SbNIP2;1 269 HKDMSQKLS 0.995 *S*

SbNIP2;1 273 SQKLSSFKL 0.597 *S*

SbNIP2;1 274 QKLSSFKLR 0.942 *S*

SbNIP2;1 282 RRLQSQSVA 0.582 *S*

SbNIP2;1 284 LQSQSVAAE 0.507 *S*

_________________________^_________________

Threonine predictions

Name Pos Context Score Pred

_________________________v_________________

SbNIP2;1 157 LGCTTPTGP 0.694 *T*

SbNIP2;1 222 NPARTLGPA 0.695 *T*

SbNIP2;1 264 EAPSTHKDM 0.863 *T*

_________________________^_________________


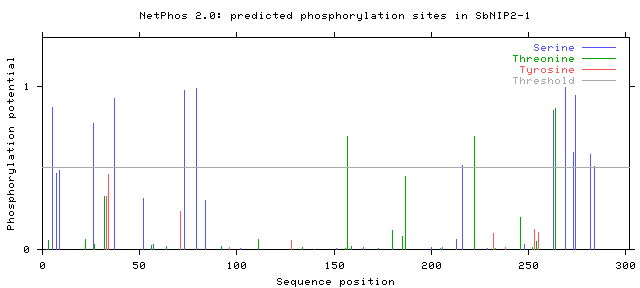


295 SbNIP2;2

MAASTASRTNSRVNYSNEIHDLSTVQSGSAVPTLFYPDKSIADIFPPHLGKKVISEVVATFLLVFVTCGAASIYGEDNKR 80

ISQLGQSVAGGLIVTVMIYATGHISGAHMNPAVTLSFACFRHFPWIQVPFYWAAQFTGAMCAAFVLKAVLHPIAVIGTTT 160

PSGPHWHALVIEIVVTFNMMFVTCAVATDSRAVGELAGLAVGSAVCITSIFAGPVSGGSMNPARTLAPAVASNVFTGLWI 240

YFLGPVIGTLSGAWVYTYIRFEEAPAAKDTQRLSSFKLRRLQSQSALAADEFDTV 320

......S...S....S............S..........S..............S................S........ 80

.S.............................................................................T 160

..........................................................S.....T............... 240

........T....................T...SS.......S............ 320

Serine predictions

Name Pos Context Score Pred

_________________________v_________________

SbNIP2;2 7 ASTASRTNS 0.917 *S*

SbNIP2;2 11 SRTNSRVNY 0.946 *S*

SbNIP2;2 16 RVNYSNEIH 0.820 *S*

SbNIP2;2 29 VQSGSAVPT 0.561 *S*

SbNIP2;2 40 YPDKSIADI 0.987 *S*

SbNIP2;2 55 KKVISEVVA 0.511 *S*

SbNIP2;2 72 CGAASIYGE 0.971 *S*

SbNIP2;2 82 NKRISQLGQ 0.992 *S*

SbNIP2;2 219 VSGGSMNPA 0.539 *S*

SbNIP2;2 274 TQRLSSFKL 0.965 *S*

SbNIP2;2 275 QRLSSFKLR 0.994 *S*

SbNIP2;2 283 RRLQSQSAL 0.673 *S*

_________________________^_________________

Threonine predictions

Name Pos Context Score Pred

_________________________v_________________

SbNIP2;2 160 IGTTTPSGP 0.963 *T*

SbNIP2;2 225 NPARTLAPA 0.550 *T*

SbNIP2;2 249 PVIGTLSGA 0.515 *T*

SbNIP2;2 270 AAKDTQRLS 0.869 *T*

_________________________^_________________


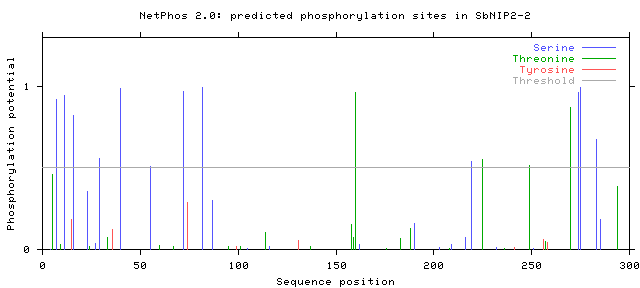


295 TaNIP2;1

MATNSRSNSRATFSSEIHDIGTVQNSTTPSMVYYTERSIADYFPPHLLKKVVSEVVSTFLLVFVTCGAAAISAHDVTRIS 80

QLGQSVAGGLIVVVMIYAVGHISGAHMNPAVTLAFAIFRHFPWIQVPFYWAAQFTGAICASFVLKAVLHPITVIGTTEPV 160

GPHWHALVIEVVVTFNMMFVTLAVATDTRAVGELAGLAVGSSVCITSIFAGAVSGGSMNPARTLGPALASNRYPGLWLYF 240

LGPVLGTLSGAWTYTYIRFEEPPKDGPQKLSSFKLRRLQSQSVAADDDELDHIPV 320

......S.S..T.S...........S.......Y...S.................................S.......S 80

................................................................................ 160

........................................................S.....T................. 240

...............................S.......S.S............. 320

Serine predictions

Name Pos Context Score Pred

_________________________v_________________

TaNIP2;1 7 TNSRSNSRA 0.720 *S*

TaNIP2;1 9 SRSNSRATF 0.510 *S*

TaNIP2;1 14 RATFSSEIH 0.932 *S*

TaNIP2;1 26 TVQNSTTPS 0.652 *S*

TaNIP2;1 38 YTERSIADY 0.912 *S*

TaNIP2;1 72 AAAISAHDV 0.780 *S*

TaNIP2;1 80 VTRISQLGQ 0.953 *S*

TaNIP2;1 217 VSGGSMNPA 0.517 *S*

TaNIP2;1 272 QKLSSFKLR 0.864 *S*

TaNIP2;1 280 RRLQSQSVA 0.582 *S*

TaNIP2;1 282 LQSQSVAAD 0.514 *S*

_________________________^_________________

Threonine predictions

Name Pos Context Score Pred

_________________________v_________________

TaNIP2;1 12 NSRATFSSE 0.571 *T*

TaNIP2;1 223 NPARTLGPA 0.695 *T*

_________________________^_________________


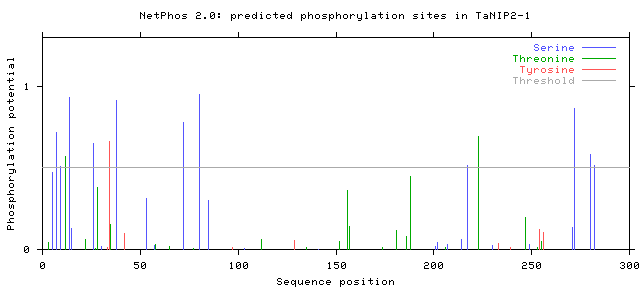


300 TaNIP2;2

MSVTSNTPTRANSRVNYSNEIHDLSTVQDGAPSLAPSMYYQEKSFADFFPPHLGKKVISEVVATFLLVFVTCGAASIYGA 80

DVTRVSQLGQSVVGGLIVTVMIYATGHISGAHMNPAVTLSFACFRHFPWIQVPFYWAAQFTGAMCAAFVLRAVLHPITVL 160

GTTTPTGPHWHALVIEIIVTFNMMFITCAVATDSRAVGELAGLAVGSAVCITSIFAGPVSGGSMNPARTLAPAVASGVYT 240

GLWIYFLGPVIGTLSGAWVYTYIRFEEEPSVKDGPQKLSSFKLRRLQSQRSMAVDEFDHV 320

....S.T.....S....S..................S......S..............S................S.... 80

.....S....S..................................................................... 160

...T..........................................................S.....T........... 240

............T................S.........S.......S..S......... 320

Serine predictions

Name Pos Context Score Pred

_________________________v_________________

TaNIP2;2 5 MSVTSNTPT 0.509 *S*

TaNIP2;2 13 TRANSRVNY 0.978 *S*

TaNIP2;2 18 RVNYSNEIH 0.820 *S*

TaNIP2;2 37 SLAPSMYYQ 0.903 *S*

TaNIP2;2 44 YQEKSFADF 0.846 *S*

TaNIP2;2 59 KKVISEVVA 0.511 *S*

TaNIP2;2 76 CGAASIYGA 0.974 *S*

TaNIP2;2 86 VTRVSQLGQ 0.973 *S*

TaNIP2;2 91 QLGQSVVGG 0.555 *S*

TaNIP2;2 223 VSGGSMNPA 0.539 *S*

TaNIP2;2 270 EEEPSVKDG 0.998 *S*

TaNIP2;2 280 QKLSSFKLR 0.864 *S*

TaNIP2;2 288 RRLQSQRSM 0.901 *S*

TaNIP2;2 291 QSQRSMAVD 0.631 *S*

_________________________^_________________

Threonine predictions

Name Pos Context Score Pred

_________________________v_________________

TaNIP2;2 7 VTSNTPTRA 0.790 *T*

TaNIP2;2 164 LGTTTPTGP 0.880 *T*

TaNIP2;2 229 NPARTLAPA 0.550 *T*

TaNIP2;2 253 PVIGTLSGA 0.515 *T*

_________________________^_________________


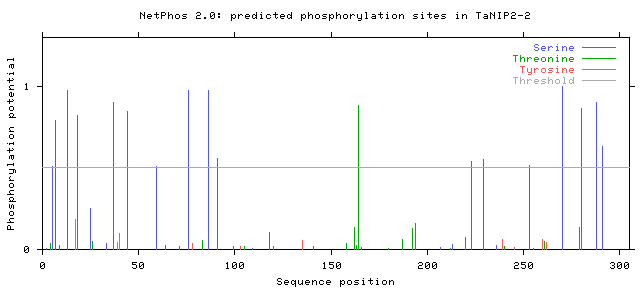


294 VvNIP2;1

MATTDPNLRSSTSINELTTALHHPDSQNSNPCFLWRLCLEHYPPGFLRKVVAEVIATYLLVFVTCGSAALSASDEQRVSK 80

LGASVAGGLMVTAMIYAVGHISGAHMNPAVTLAFAAVRHFPWKQVPLYAAAQLTGAIGAAFTLRELLYPIKHLGTTTPSG 160

TEIQALVMEIVVTFSMMFITSAVATDTKAIGELAGIAVGSAVCITSILAGPVSGGSMNPARTLGPAIASADYKGIWVYAV 240

GPVSGTLLGTWSYNFIRVTEKPVQAISPHSFSLKLCRMRSNAGEISSKDPLNHV 320

..........S.S............S............................................S.S.....S. 80

.............................................................T.....Y........T.S. 160

.......................................................S.....T.................. 240

..................T.......S....S.............S........ 320

Serine predictions

Name Pos Context Score Pred

_________________________v_________________

VvNIP2;1 11 NLRSSTSIN 0.991 *S*

VvNIP2;1 13 RSSTSINEL 0.913 *S*

VvNIP2;1 26 HHPDSQNSN 0.627 *S*

VvNIP2;1 71 SAALSASDE 0.992 *S*

VvNIP2;1 73 ALSASDEQR 0.696 *S*

VvNIP2;1 79 EQRVSKLGA 0.964 *S*

VvNIP2;1 159 TTTPSGTEI 0.880 *S*

VvNIP2;1 216 VSGGSMNPA 0.539 *S*

VvNIP2;1 267 VQAISPHSF 0.836 *S*

VvNIP2;1 272 PHSFSLKLC 0.548 *S*

VvNIP2;1 286 AGEISSKDP 0.988 *S*

_________________________^_________________

Threonine predictions

Name Pos Context Score Pred

_________________________v_________________

VvNIP2;1 142 GAAFTLREL 0.975 *T*

VvNIP2;1 157 LGTTTPSGT 0.967 *T*

VvNIP2;1 222 NPARTLGPA 0.695 *T*

VvNIP2;1 259 FIRVTEKPV 0.950 *T*

_________________________^_________________


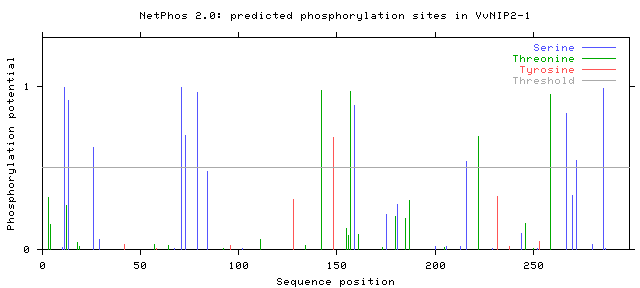


295 ZmNIP2;1

MSTNSRSNSRANFNNEIHDIGTAQNSSMPPTYYDRSLADIFPPHLLKKVVSEVVSTFLLVFVTCGAAGIYGSDKDRISQL 80

GQSVAGGLIVTVMIYAVGHISGAHMNPAVTLAFAVFRHFPWIQVPFYWAAQFTGSICASFVLKAVLHPIAVLGTTTPTGP 160

HWHSLVIEIIVTFNMMFVTLAVATDTRAVGELAGLAVGSAVCITSIFAGAVSGGSMNPARTLGPALASNLYTGLWIYFLG 240

PVLGTLSGAWTYTYIRFEEAPSHKDMSQKLSSFKLRRLQSQSVAVDDDELDHIQV 320

....S.....................S........S...................................S.....S.. 80

...........................................................................T.... 160

......................................................S.....T................... 240

.....................S....S...SS.......S............... 320

Serine predictions

Name Pos Context Score Pred

_________________________v_________________

ZmNIP2;1 5 MSTNSRSNS 0.871 *S*

ZmNIP2;1 27 AQNSSMPPT 0.567 *S*

ZmNIP2;1 36 YYDRSLADI 0.926 *S*

ZmNIP2;1 72 GIYGSDKDR 0.974 *S*

ZmNIP2;1 78 KDRISQLGQ 0.987 *S*

ZmNIP2;1 215 VSGGSMNPA 0.517 *S*

ZmNIP2;1 262 EEAPSHKDM 0.998 *S*

ZmNIP2;1 267 HKDMSQKLS 0.992 *S*

ZmNIP2;1 271 SQKLSSFKL 0.597 *S*

ZmNIP2;1 272 QKLSSFKLR 0.942 *S*

ZmNIP2;1 280 RRLQSQSVA 0.717 *S*

_________________________^_________________

Threonine predictions

Name Pos Context Score Pred

_________________________v_________________

ZmNIP2;1 156 LGTTTPTGP 0.880 *T*

ZmNIP2;1 221 NPARTLGPA 0.695 *T*

_________________________^_________________


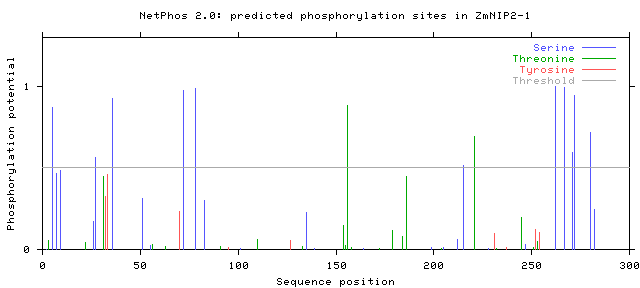


294 ZmNIP2;2

MAAASTTSRTNSRVNYSNEIHDLSTVQSGSVVPTLFYPDKSIADIFPPHLGKKVISEVVATFLLVFVTCGAASIYGEDNR 80

RISQLGQSVAGGLIVTVMIYATGHISGAHMNPAVTLSFACFRHFPWIQVPFYWAAQFTGAMCAAFVLKAVLHPIAVIGTT 160

TPSGPHWHALLIEIVVTFNMMFVTCAVATDSRAVGELAGLAVGSAVCITSIFAGPVSGGSMNPARTLAPAVASNVFTGLW 240

IYFLGPVIGTLSGAWVYTYIRFEEAPAAKDTQRLSSFKLRRMQSQLAADEFDTV 320

.....TTS...S....S............S..........S..............S................S....... 80

..S............................................................................. 160

T..........................................................S.....T.............. 240

.........T....................T...SS.......S.......... 320

Serine predictions

Name Pos Context Score Pred

_________________________v_________________

ZmNIP2;2 8 ASTTSRTNS 0.959 *S*

ZmNIP2;2 12 SRTNSRVNY 0.988 *S*

ZmNIP2;2 17 RVNYSNEIH 0.820 *S*

ZmNIP2;2 30 VQSGSVVPT 0.817 *S*

ZmNIP2;2 41 YPDKSIADI 0.987 *S*

ZmNIP2;2 56 KKVISEVVA 0.511 *S*

ZmNIP2;2 73 CGAASIYGE 0.971 *S*

ZmNIP2;2 83 NRRISQLGQ 0.996 *S*

ZmNIP2;2 220 VSGGSMNPA 0.539 *S*

ZmNIP2;2 275 TQRLSSFKL 0.965 *S*

ZmNIP2;2 276 QRLSSFKLR 0.994 *S*

ZmNIP2;2 284 RRMQSQLAA 0.538 *S*

_________________________^_________________

Threonine predictions

Name Pos Context Score Pred

_________________________v_________________

ZmNIP2;2 6 AAASTTSRT 0.646 *T*

ZmNIP2;2 7 AASTTSRTN 0.642 *T*

ZmNIP2;2 161 IGTTTPSGP 0.963 *T*

ZmNIP2;2 226 NPARTLAPA 0.550 *T*

ZmNIP2;2 250 PVIGTLSGA 0.515 *T*

ZmNIP2;2 271 AAKDTQRLS 0.869 *T*

_________________________^_________________


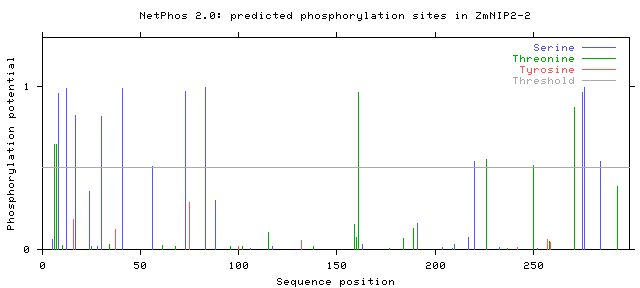


301 ZmNIP2;3

MAASTTSRTNSRVNYSNEIHDLSTVQGGSAAAAAAALFYPDSKSIADIFPPHLGKKVISEVVATFLLVFVTCGAASIYGE 80

DNARISQLGQSVAGGLIVTVMIYATGHISGAHMNPAVTLSFACFRHFPWIQVPFYWAAQFTGAMCAAFVLKAVLQPIAVI 160

GTTTPSGPHWHALAIEIVVTFNMMFVTCAVATDSRAVGELAGLAVGSAVCITSIFAGPVSGGSMNPARTLAPAVASNVFT 240

GLWIYFLGPVVGTLSGAWVYTYIRFEEAPAAAKPDTQRLSSFKLRRMQSQSALAADEFDTV 320

....TTS...S....S...........................S..............S................S.... 80

.....S.......................................................................... 160

...T..........................................................S.....T........... 240

............T......................T...SS.......S............ 320

Serine predictions

Name Pos Context Score Pred

_________________________v_________________

ZmNIP2;3 7 ASTTSRTNS 0.959 *S*

ZmNIP2;3 11 SRTNSRVNY 0.988 *S*

ZmNIP2;3 16 RVNYSNEIH 0.820 *S*

ZmNIP2;3 44 PDSKSIADI 0.973 *S*

ZmNIP2;3 59 KKVISEVVA 0.511 *S*

ZmNIP2;3 76 CGAASIYGE 0.971 *S*

ZmNIP2;3 86 NARISQLGQ 0.974 *S*

ZmNIP2;3 223 VSGGSMNPA 0.539 *S*

ZmNIP2;3 280 TQRLSSFKL 0.965 *S*

ZmNIP2;3 281 QRLSSFKLR 0.994 *S*

ZmNIP2;3 289 RRMQSQSAL 0.657 *S*

_________________________^_________________

Threonine predictions

Name Pos Context Score Pred

_________________________v_________________

ZmNIP2;3 5 MAASTTSRT 0.686 *T*

ZmNIP2;3 6 AASTTSRTN 0.642 *T*

ZmNIP2;3 164 IGTTTPSGP 0.963 *T*

ZmNIP2;3 229 NPARTLAPA 0.550 *T*

ZmNIP2;3 253 PVVGTLSGA 0.513 *T*

ZmNIP2;3 276 AKPDTQRLS 0.822 *T*

_________________________^_________________


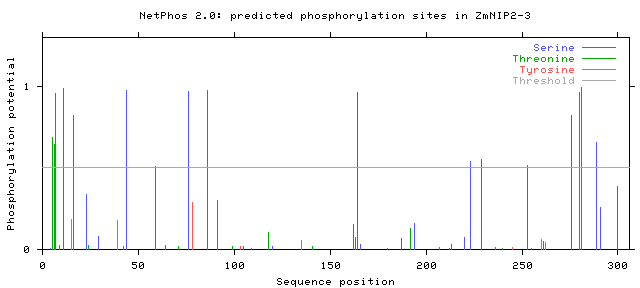

Supplement: Additional file 7 — The predicted serine and threonine phosphorylation sites in plant NIP III proteins. [file 1471-2229-10-256-S7.DOC]
