# Supplementary material for: Functional divergence of the NIP III subgroup proteins involved altered selective constraints and positive selection
Source: BMC Plant Biol. 2010 Nov 20;10:256. doi: 10.1186/1471-2229-10-256 (PMC3095335; doi:10.1186/1471-2229-10-256)
Supplement: Additional file 8 — Significantly Type-II functional divergence related amino acid sites in grasses. The six putative transmembrane regions (TMs) are shadowed in black. The 7 amino acid candidates identified responsible for the Type-II functional divergence between NIP2;1 and NIP2;2 proteins are highlighted in red. [file 1471-2229-10-256-S8.DOC]

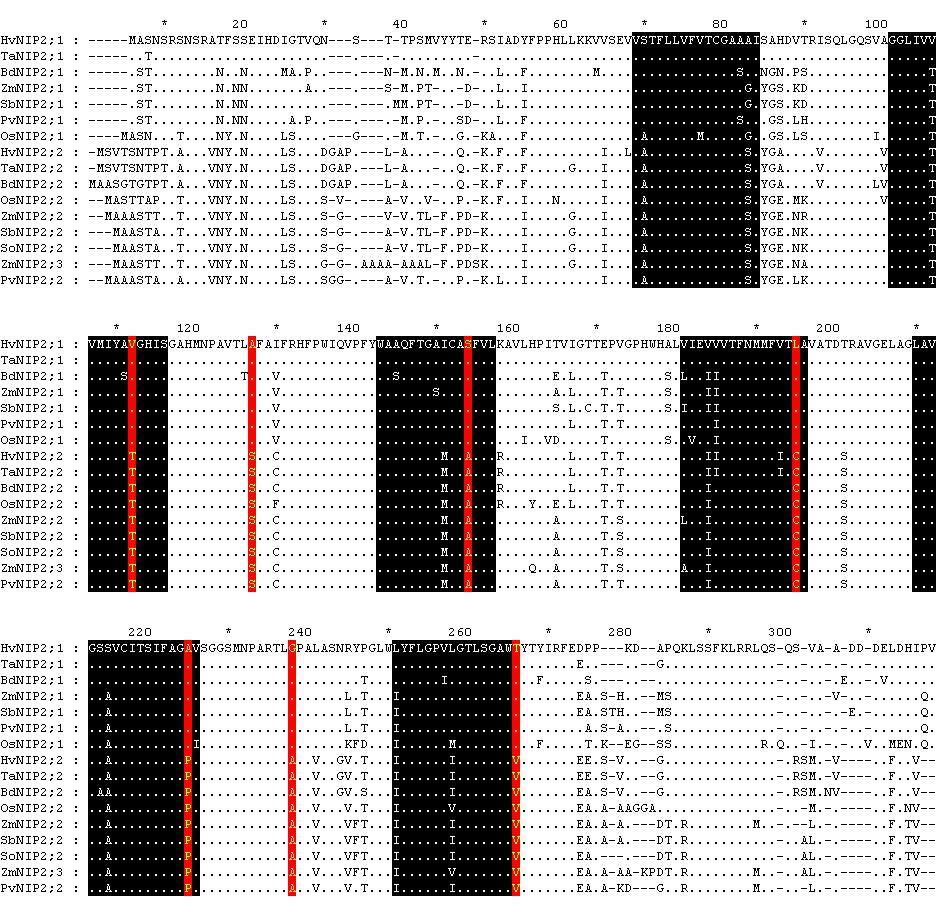


**Additional file 8**

Significantly Type-II functional divergence related amino acid sites in grasses. The six putative transmembrane regions (TMs) are shadowed in black. The 7 amino acid candidates identified responsible for the Type-II functional divergence between NIP2;1 and NIP2;2 proteins are highlighted in red.
