# Supplementary material for: Functional divergence of the NIP III subgroup proteins involved altered selective constraints and positive selection
Source: BMC Plant Biol. 2010 Nov 20;10:256. doi: 10.1186/1471-2229-10-256 (PMC3095335; doi:10.1186/1471-2229-10-256)
Supplement: Additional file 9 — Simulated structures of rice OsNIP2;1 (A-B) and cucumber CsNIP2;1 (C-D). Specificity-determining positions (SDPs) are colored in blue. The possible SDP 84Q (OsNIP2;1) or 83A (CsNIP2;1) is circled and designated with arrow. The NPA motifs and ar/R filter are highlighted in green and red respectively. Positively selected sites and significantly Type-I functional divergence related sites are shaded in yellow and purple respectively. [file 1471-2229-10-256-S9.DOC]

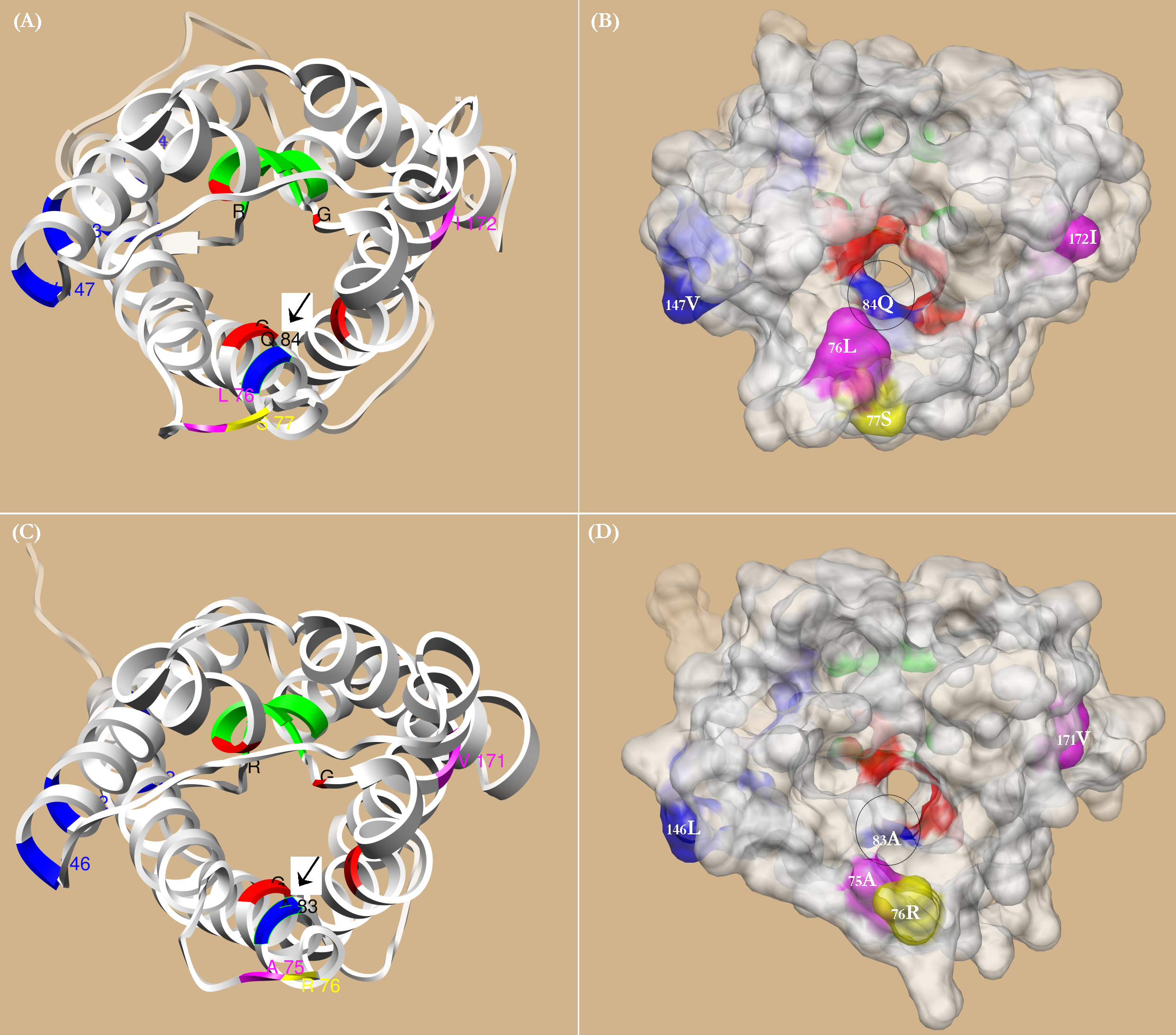


**Additional file 9**

Simulated structures of rice OsNIP2;1 (A-B) and cucumber CsNIP2;1 (C-D). Specificity-determining positions (SDPs) are colored in blue. The possible SDP 84Q (OsNIP2;1) or 83A (CsNIP2;1) is circled and designated with arrow. The NPA motifs and ar/R filter are highlighted in green and red respectively. Positively selected sites and significantly Type-I functional divergence related sites are shaded in yellow and purple respectively.
